# Prognostic value of long non-coding RNA *MALAT1* in hepatocellular carcinoma: A study based on multi-omics analysis and RT-PCR validation

**DOI:** 10.3389/pore.2022.1610808

**Published:** 2023-01-04

**Authors:** Xiaoli Liao, Junming Chen, DongCheng Luo, Baohua Luo, Wenfeng Huang, Weimin Xie

**Affiliations:** ^1^ Department of Chemotherapy, Guangxi Medical University Cancer Hospital, Nanning, China; ^2^ Department of Medical Oncology, Fujian Medical University Union Hospital, Fuzhou, China; ^3^ Department of Gastroenterology, Jiangbin Hospital, Nanning, China; ^4^ Department of Medical Oncology, Second Affiliated Hospital of Guangxi Medical University, Nanning, China

**Keywords:** prognosis, hepatocellular carcinoma, mutation, DNA methylation, metastasis associated lung adenocarcinoma transcript 1 (*MALAT1*), interacting proteins, competing endogenous RNAs (ceRNAs), multi-omics

## Abstract

**Background:** This study aimed to explore the relationship between *MALAT1* and the prognosis of patients with hepatocellular carcinoma (HCC).

**Methods:** We constructed a *MALAT1* protein-protein interaction network using the STRING database and a network of competing endogenous RNAs (ceRNAs) using the StarBase database. Using data from the GEPIA2 database, we studied the association between genes in these networks and survival of patients with HCC. The potential mechanisms underlying the relationship between *MALAT1* and HCC prognosis were studied using combined data from RNA sequencing, DNA methylation, and somatic mutation data from The Cancer Genome Atlas (TCGA) liver cancer cohort. Tumor tissues and 19 paired adjacent non-tumor tissues (PANTs) from HCC patients who underwent radical resection were analyzed for *MALAT1* mRNA levels using real-time PCR, and associations of *MALAT1* expression with clinicopathological features or prognosis of patients were analyzed using log-rank test and Gehan-Breslow-Wilcoxon test.

**Results:** Five interacting proteins and five target genes of *MALAT1* in the ceRNA network significantly correlated with poor survival of patients with HCC (*p* < 0.05). High *MALAT1* expression was associated with mutations in two genes leading to poor prognosis and may upregulate some prognostic risk genes through methylation. *MALAT1* was significantly co-expressed with various signatures of genes involved in HCC progression, including the cell cycle, DNA damage repair, mismatch repair, homologous recombination, molecular cancer m6A, exosome, ferroptosis, infiltration of lymphocyte (*p* < 0.05). The expression of *MALAT1* was markedly upregulated in HCC tissues compared with PANTs. In Kaplan-Meier analysis, patients with high *MALAT1* expression had significantly shorter progression-free survival (PFS) (*p* = 0.033) and overall survival (OS) (*p* = 0.023) than those with low *MALAT1* expression. Median PFS was 19.2 months for patients with high *MALAT1* expression and 52.8 months for patients with low expression, while the corresponding median OS was 40.5 and 78.3 months. In subgroup analysis of patients with vascular invasion, cirrhosis, and HBsAg positive or AFP positive, MALAT1 overexpression was significantly associated with shorter PFS and OS. Models for predicting PFS and OS constructed based on *MALAT1* expression and clinicopathological features had moderate predictive power, with areas under the receiver operating characteristic curves of 0.661–0.731. Additionally, *MALAT1* expression level was significantly associated with liver cirrhosis, vascular invasion, and tumor capsular infiltration (*p* < 0.05 for all).

**Conclusion:** *MALAT1* is overexpressed in HCC, and higher expression is associated with worse prognosis. *MALAT1* mRNA level may serve as a prognostic marker for patients with HCC after hepatectomy.

## Introduction

Hepatocellular carcinoma (HCC) is the most frequent subtype of primary liver cancer and a common malignancy. HCC has been rising in incidence in the last decades and is a major cancer-related cause of death among men in China (1, 2). Non-curative therapies such as transcatheter arterial chemoembolization (TACE) and stereotactic radiotherapy (SBRT) can prolong the survival of patients with advanced HCC by slowing tumor progression (3, 4), while the combination of angiogenesis and immune checkpoint inhibitors can prolong progression-free survival (PFS) (5, 6).

Patients with early disease can benefit from curative treatments such as radical resection, liver transplantation (LT), and ablative techniques (7–9), but 5-year recurrence rates after such procedures are 40%–70% (8). A growing body of studies has focused on screening and clinical application of biomarkers for prognostic monitoring, but the sensitivity and specificity of traditional prognostic indicators such as alpha-fetoprotein (AFP) are inadequate on their own to guide clinical decisions (9). Emerging markers based on histology and genomics may more comprehensively reflect the biological characteristics of cancer cells and therefore support individualized diagnosis and treatment of HCC (10).

Genomic and transcriptomic sequencing studies suggest that long non-coding RNAs (lncRNAs) regulate many of the characteristics of HCC, including proliferation, invasion, metastasis, and immunosuppression (11, 12). These RNAs more than 200 nucleotides long are not translated into proteins but can regulate gene expression at the levels of transcription, post-transcriptional processing, and translation (13). In addition, lncRNAs may compete with microRNAs (miRNAs) to bind to the same target mRNA in order to regulate its translation (14). In this case, the lncRNA is referred to as a competing endogenous RNA (ceRNA). LncRNAs can also regulate DNA methylation to influence cancer (15, 16).

Recently, studies have shown that the 8-kb lncRNA *MALAT1* could be linked to other cancers, including breast cancer, prostate cancer, pancreatic cancer, glioma, and leukemia. (17). Therefore, *MALAT1* may be a biomarker for early diagnosis, severity assessment, or prognostic evaluation in HCC (18). In this study, we analyzed *MALAT1* expression at the multi-omics level and identified associations of its expression with gene transcription, DNA methylation, and gene mutations, and we explored the potential influence of these associations on prognosis. Second, we investigated the relationships of *MALAT1* expression with clinicopathological features and prognosis of HCC patients after hepatectomy.

## Methods

### Public data preparation and preprocessing

RNA-sequencing (RNA-seq) counts and tumor data for 368 patients in The Cancer Genome Atlas liver hepatocellular carcinoma (TCGA-LIHC) cohort were downloaded using UCSCXenaTools (19). Counts were normalized using the transcript per million (TPM) method, and genes with expression levels below the lower end of the interquartile range (IQR) of expression across all genes in the sample were excluded. Samples with low-frequency counts (the number of occasions of “count-per-million values > 0.5” in the sample less than 2) were excluded.

We also downloaded somatic mutation and DNA methylation profiling data for 364 and 430 patients, respectively, in the TCGA-LIHC cohort using UCSCXenaTools.

### Interacting protein analysis

We constructed the network of *MALAT1* with interacting proteins using text mining in the STRING database. A first shell was created with no more than 10 interactors, and a second shell with no more than 20 interactors. Enrichment of terms from the Kyoto Encyclopedia of Genes and Genomes (KEGG) in the network were visualized using the GOplot package in R. Genes encoding first-shell proteins were analyzed for potential assocations with HCC patient survival using the GEPIA2 database (http://gepia2.cancer-pku.cn/).

### Construction of ceRNA network

StarBase is an online database that can build ceRNA networks based on thousands of interactions between miRNAs and their target genes in a CLIP-seq (Crosslinking or RNA immunoprecipitation followed by sequencing) database (https://starbase.sysu.edu.cn/). We obtained potential *MALAT1* target miRNAs and genes that may compete with *MALAT1* for binding miRNAs through StarBase (screening by miRNA number ≥ 30 and *p*-value ≤ 1.0e-5). In this way, a network of ceRNAs was constructed and visualized by Cytoscape. Genes regulated by *MALAT1* and miRNAs were analyzed for their potential associations with survival of HCC patients using the GEPIA2 database.

### Somatic mutation data analysis

Somatic mutations were analyzed using the maftools package in R (4.1.0). We selected samples tested for both expression and mutation, then selected 84 samples whose *MALAT1* expression was below the lower end of the IQR and 85 samples whose *MALAT1* expression exceeded the upper end of the IQR. We summarized the overall mutation profiles of samples expressing low or high *MALAT1,* then we compared the prevalences of differentially mutated genes between the two groups using Fisher’s exact test and enrichment analysis. Differences associated with *p* < 0.05 were considered significant. Samples were categorized as wild-type or mutant depending on the presence or absence of somatic mutations. Survival of patients in the wild-type or mutant groups was compared using the Kaplan-Meier method and log-rank test.

### DNA methylation data analysis

After being loaded, methylation data were filtered, quality-controlled, and normalized using the ChAMP package in R. Beta values were the quantification values of methylation levels. The singular value decomposition method (SVD) calculated the correlation between biological factors (*MALAT1* and other clinical features) and the variation of beta values. Differentially methylated positions (DMPs) were identified by comparing samples expressing high or low *MALAT1*. We considered that the following DMPs could upregulate target genes of *MALAT1*: hypermethylated DMPs in the coding sequence of the gene, or hypomethylated DMPs in the promoter or transcriptional start site (TSS). Conversely, we considered that the following DMPs could downregulate target genes of *MALAT1:* hypomethylated DMPs in the coding sequence of the gene, or hypermethylated DMPs in the promoter or TSS. We then calculated correlation coefficients between *MALAT1* and target genes to identify genes regulated by *MALAT1*-associated methylation. Results associated with *p* < 0.05 were considered significant. Cox regression was performed on the beta values of each DMP. DMPs that emerged as significant (*p* < 0.05) were shown as forest plots. Enrichment of Molecular Signatures Database’s terms or pathways in DMPs was assessed using gene set enrichment analysis (GSEA) using the “champ.GSEA()” function.

### Analysis of tumor-associated signatures and patient survival

We used the Immuno-Oncology-Biological-Research (IOBR) package in R integrating six commonly used algorithms (MCPcounter, TIMER, xCell, CIBERSORT, EPIC, and quanTiseq), to calculate scores for tumor-infiltrating lymphocytes (TILs) and tumor-associated signatures. Scores that reached statistical significance were determined for 92 samples whose *MALAT1* expression was below the lower end of the IQR and 92 samples whose *MALAT1* expression exceeded the upper end of the IQR. Kaplan-Meier analysis was used to compare survival of patients whose *MALAT1* expression was above or below the median, based on data for the TCGA-LIHC cohort. Differences between the two groups were compared using the log-rank test.

### Collection of clinical data and survival information

We analyzed samples from 179 HCC patients who underwent radical resection in the Affiliated Tumor Hospital of Guangxi Medical University between April 2004 and December 2012. None of the patients received TACE, radiotherapy, chemotherapy, or other anti-tumor treatments before surgery. Tumor tissues from all patients, as well as paired adjacent non-tumor tissues (PANTs) from 19 patients, were frozen in liquid nitrogen immediately after surgery and stored at −80°C until RNA extraction. Two pathologists participated in the diagnosis of histopathology of resected tumor samples. One of these two pathologists is responsible for routine pathological diagnosis in the clinical practice, while the other is responsible for routine quality control in the clinical study. This work was approved by the Institutional Ethics Committee of the Affiliated Tumor Hospital of Guangxi Medical University (Nanning, China, Approval Number: LW2022145), which waived the requirement for informed consent because patients, at the time of treatment, consented for their anonymized data to be analyzed and published for research purposes.

Clinicopathological data on all patients were collected, including age, sex, Child-Pugh classification, tumor size, tumor numbers, clinical TNM stage, the presence or absence of vascular invasion, capsular infiltration, and cirrhosis. Laboratory results were also collected, such as serum levels of alpha-fetoprotein (AFP) and hepatitis B surface antigen (HBsAg).

Follow-up information of patients was collected for survival analysis. Patients were followed up every 3 months during the first 2 years, and thereafter every 6 months, for 2–10 years. Tumor recurrence was detected based on radiology and serum AFP examination. Relationships of *MALAT1* expression with PFS and overall survival (OS) were explored.

### Detection of *MALAT1* expression

Total RNA was extracted from HCC tumors and PANTs using TRIzol (Invitrogen, Carlsbad, CA, United States) and stored at −80°C. RNA (1 μg) was reverse-transcribed into complementary DNA (cDNA) using the PrimeScriptTM RT Reagent Kit (Takara Bio, Kusatsu, Japan) in reactions with a total volume of 20 μl. Real-time PCR (RT-PCR) was performed in 10-μl reactions on a LightCycler^®^ 480 II Real-time PCR system (Roche, Basel, Switzerland) using GAPDH as an internal reference. Reactions were performed in a microassay plate (Roche) at 95°C for 10 min, followed by 40 cycles at 95°C for 10 s and 60°C for 30 s. Primer sequences were: *MALAT1* forward, 5′-CAG​GCG​TTG​TGC​GTA​GAG​GA-3′; *MALAT1* reverse, 5′-TGC​CGA​CCT​CAC​GGA​TTT​T-3′; GAPDH forward, 5′-GTC​AGC​CGC​ATC​TTC​TTT-3′; and GAPDH reverse, 5′-CGC​CCA​ATA​CGA​CCA​AAT-3′. All samples were tested in triplicate. Expression levels of *MALAT1* were normalized to those of GAPDH and were calculated using the 2^−ΔΔCT^ method (20).

### Statistical analysis

All statistical analyses were performed using SPSS 22.0 (IBM, Chicago, IL, United States) and R version 4.1.0. Differences in continuous variables were compared using Student’s t-test for normally distributed data or Mann-Whitney *U*-test for skewed data. Patients’ survival was analyzed by the Kaplan-Meier method, and differences were assessed for significance using the log-rank test. Univariate analysis was performed to identify clinicopathological features associated with survival, and results were assessed for significance using the Gehan-Breslow-Wilcoxon test.

Covariates that were associated with *p* < 0.05 in univariate analysis were included in multivariate Cox analysis, which was used to select features for constructing Cox proportional hazard models to predict survival. Models for predicting PFS and OS were assessed using the area under time-dependent receiver operating characteristic curves (AUC), as calculated with the timeROC package in R. Net benefits of models was assessed using decision curves, plotted with the ggDCA package in R. The ability of *MALAT1* expression to diagnose clinicopathological features of HCC patients was assessed using AUCs. All hypothesis testing was two-sided, with *p* < 0.05 considered statistically significant.

## Results

### *MALAT1*-associated interacting proteins are associated with HCC and prognosis

**FIGURE 1:**
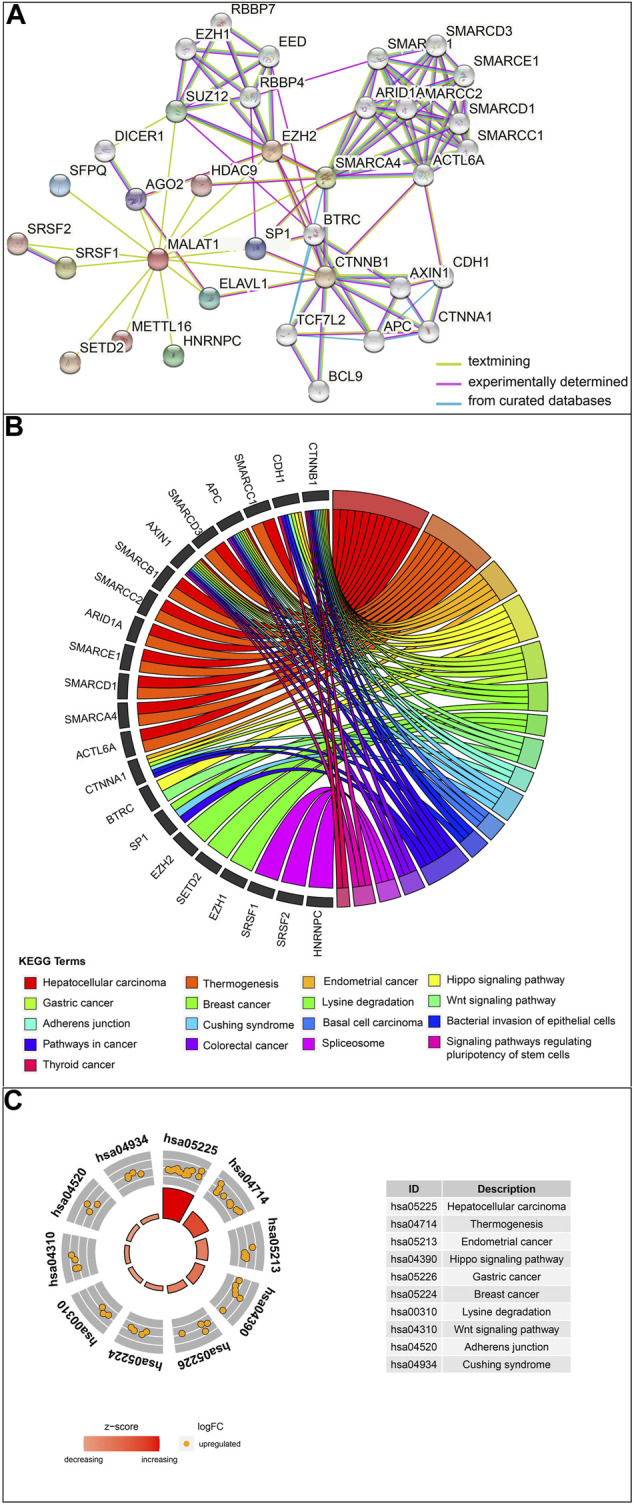
Analysis of *MALAT1*-interacting proteins. **(A)** The protein–protein interaction network of *MALAT1* was analyzed using the STRING tool. **(B)** Chord plot displaying the relationship between MALAT1-interacting proteins and Kyoto Encyclopedia of Genes and Genomes (KEGG) terms. **(C)** The outer circle shows a scatter plot for each term of the log (fold change) of the assigned genes. Orange circles indicate upregulation. The z-score is a crude measure of significance of enrichment.

**FIGURE 2:**
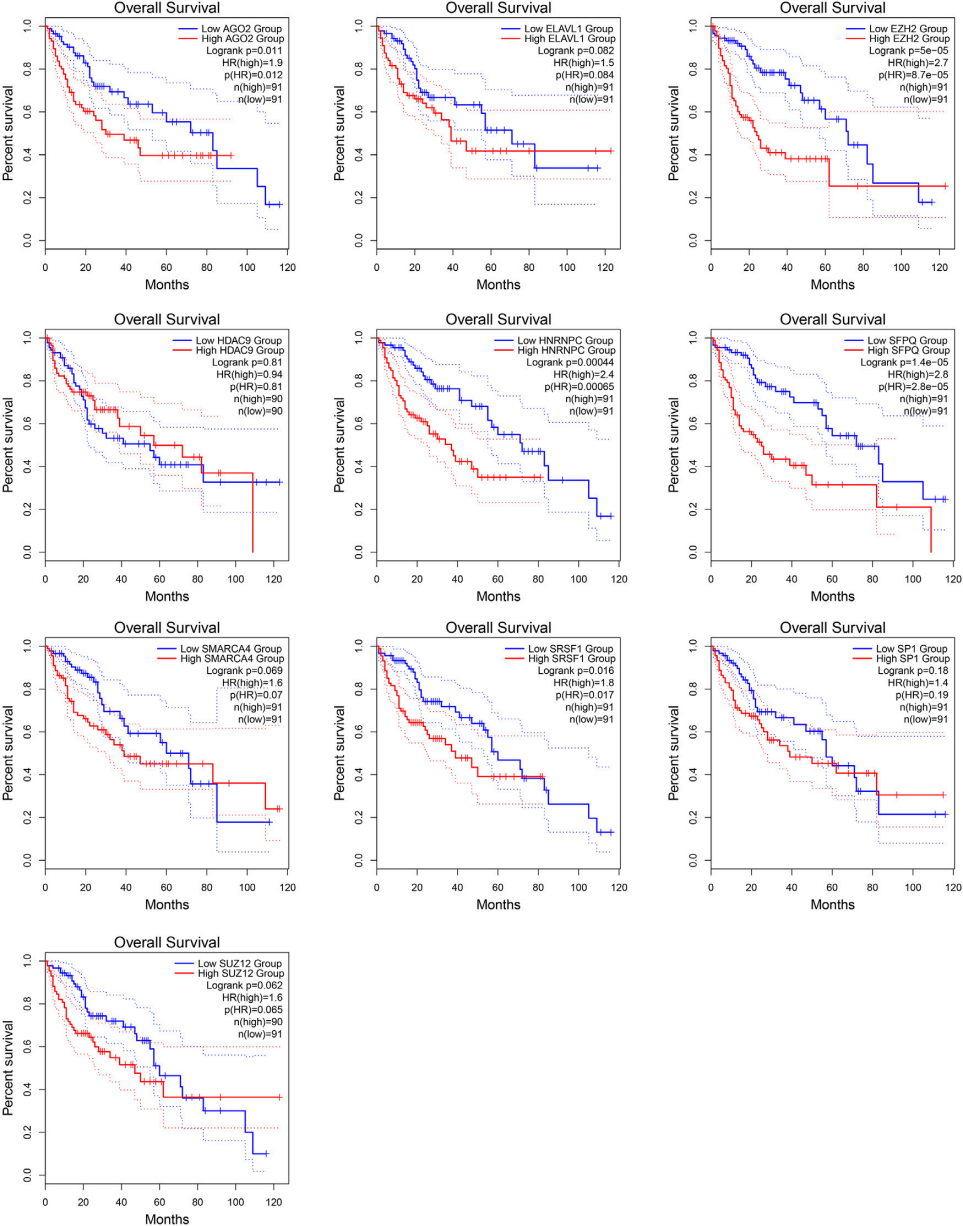
Kaplan-Meier survival curves for patients showing high or low expression of genes encoding *MALAT1*-interacting proteins.

The protein interaction network related to *MALAT1* was obtained using the text mining algorithm in *STRING* (Figure 1A). The text mining identified 10 proteins interacting with *MALAT1* as *EZH2, SRSF1, SMARCA4, HNRNPC, SUZ12, ELAVL1, SFPQ, SP1, AGO2, HDAC9*. The proteins in the network were most enriched in KEGG terms associated with cancer (Figure 1B). Among these terms, HCC had the highest z-score, indicating that the proteins in this network were most closely related to HCC (Figure 1C). Five protein-coding genes in the first shell of the network were significantly associated with worse survival when highly expressed: *AGO2, HNRNPC, EZH2, SFPQ,* and *SRSF1* (Figure 2; log-rank *p* < 0.05).

### Target genes of *MALAT1* in the ceRNA network are associated with prognosis

**FIGURE 3:**
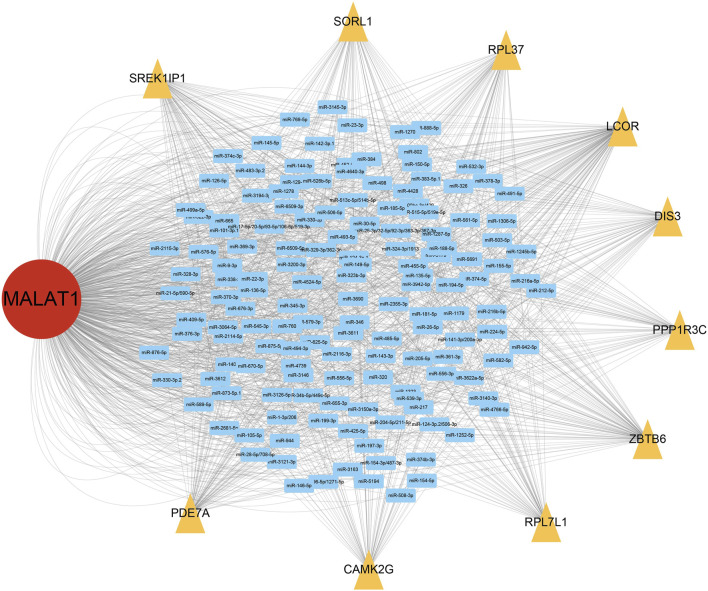
*MALAT1*-associated network of competing endogenous RNAs (ceRNAs). Blue rectangles represent microRNAs (miRNAs) that can bind to *MALAT1*; yellow triangles represent target genes that compete with *MALAT1* for binding to miRNAs.

**FIGURE 4:**
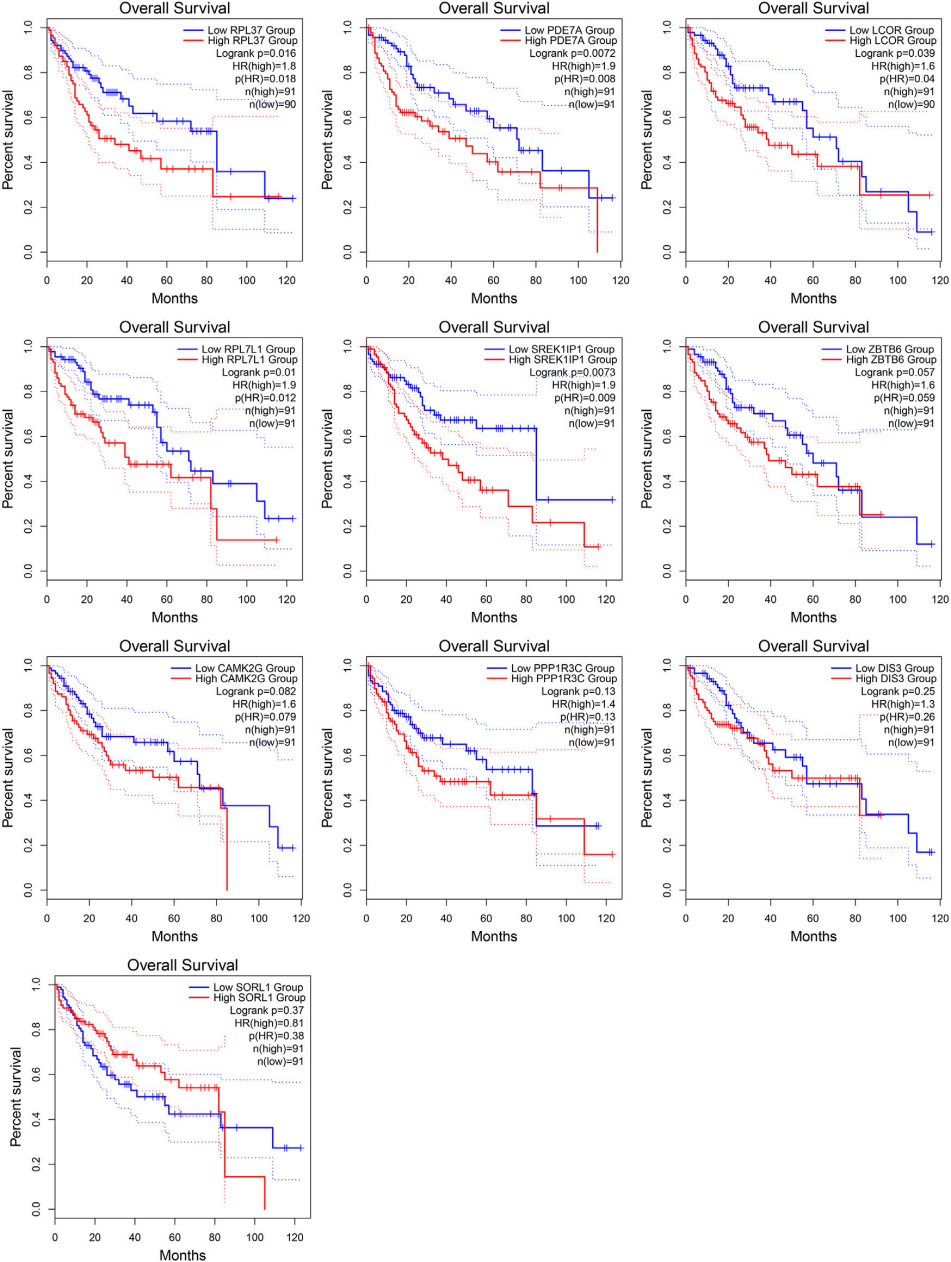
Kaplan-Meier survival curves for patients showing high or low expression of *MALAT1* target genes.

From the ceRNA network for *MALAT1*, we obtained 10 target genes: *ZBTB6, LCOR, PDE7A, SORL1, RPL37, RPL7L1, SREK1IP1, DIS3, PPP1R3C, CAMK2G*. All these genes may competitively bind to the same miRNAs as *MALAT1* (Figure 3). Higher expression of *LCOR, PDE7A, RPL7L1, RPL37,* and *SREK1IP1* was associated with worse survival of HCC patients (log-rank *p* < 0.05; Figure 4).

### *MALAT1* expression is associated with genetic mutations in HCC

**FIGURE 5:**
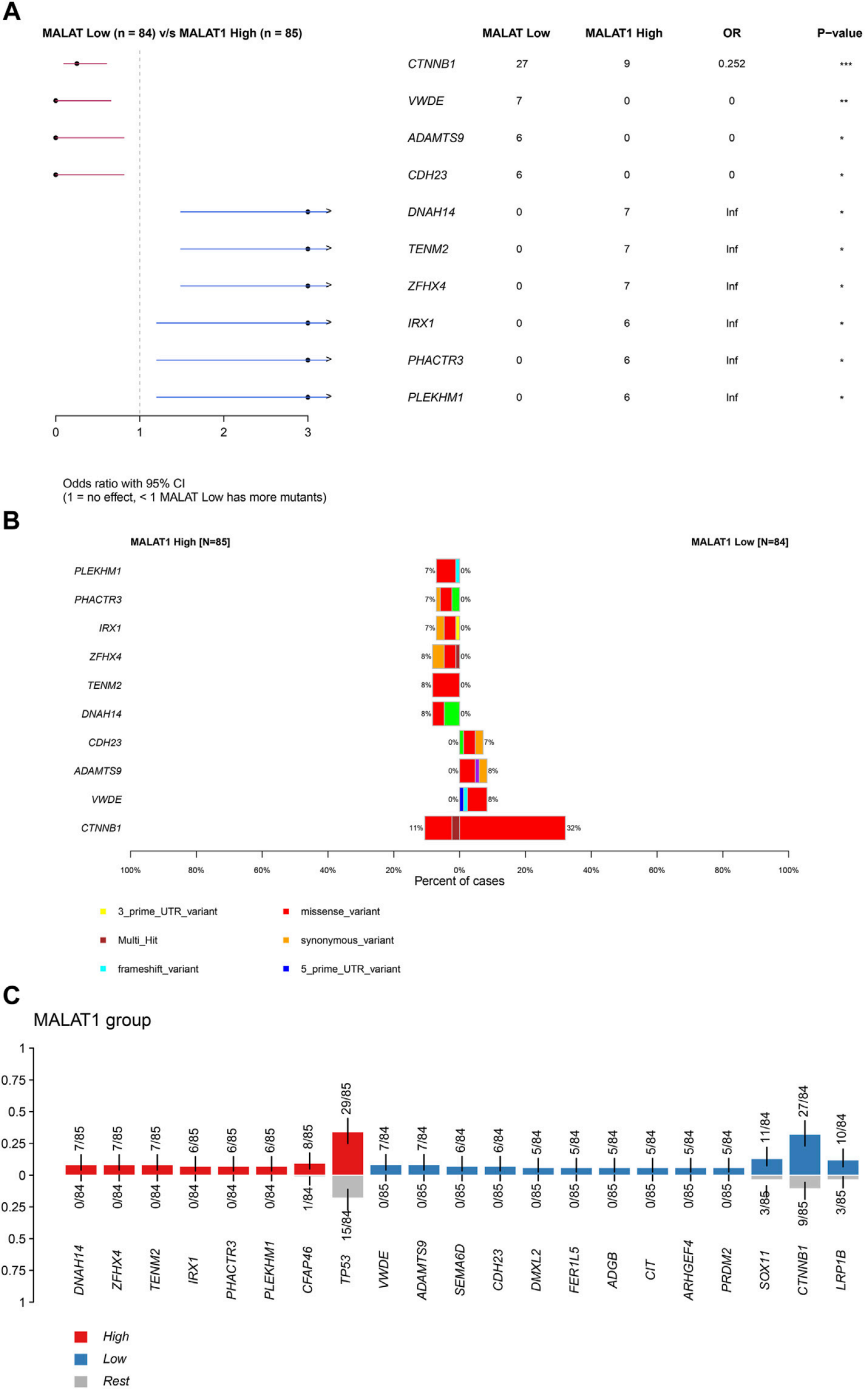
Differentially mutated genes. **(A)** Forest plot of Fisher testing to identify differentially mutated genes. **(B)** Proportion of each type of mutation and the overall mutation percentage of differentially mutated genes. The left half of the graph shows samples expressing high MALAT1 and the right half shows samples expressing low MALAT1. **(C)** Red represents mutated genes enriched in samples expressing high *MALAT1.* Blue represents mutated genes enriched in samples expressing low *MALAT1.*

**FIGURE 6:**
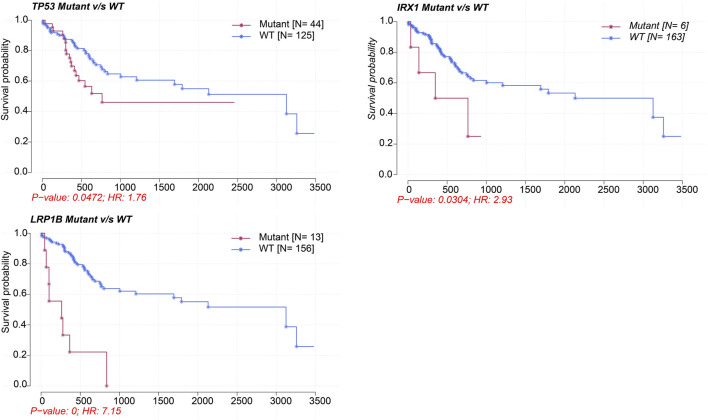
Kaplan-Meier survival curves comparing patients carrying mutant or wild-type versions of *TP53, IRX1,* and *LRP1B*.

Supplementary Figure S1 showed the mutation profiles of the samples. Samples expressing high or low *MALAT1* were compared using Fisher’s exact test to identify 10 differentially mutated genes (Figures 5A, B). Eight mutated genes were enriched in samples expressing high *MALAT1* and 13 mutated genes were enriched in samples expressing low *MALAT1* (Figure 5C). Patients with higher *MALAT1* expression showed more mutations in *IRX1* or *TP53* and mutations in IRX1 and TP53 indicated worse survival than those with the corresponding wild-type alleles (log-rank *p* < 0.05; Figure 6). Patients with lower *MALAT1* expression were associated with more *LRP1B* mutations and *LRP1B* mutations indicated worse survival (log-rank *p* < 0.05; Figure 6). Mutations in the 10 differentially mutated genes altered the protein sequences (Supplementary Figure S2).

### *MALAT1* expression is a correlate of DNA methylation in HCC

**FIGURE 7:**
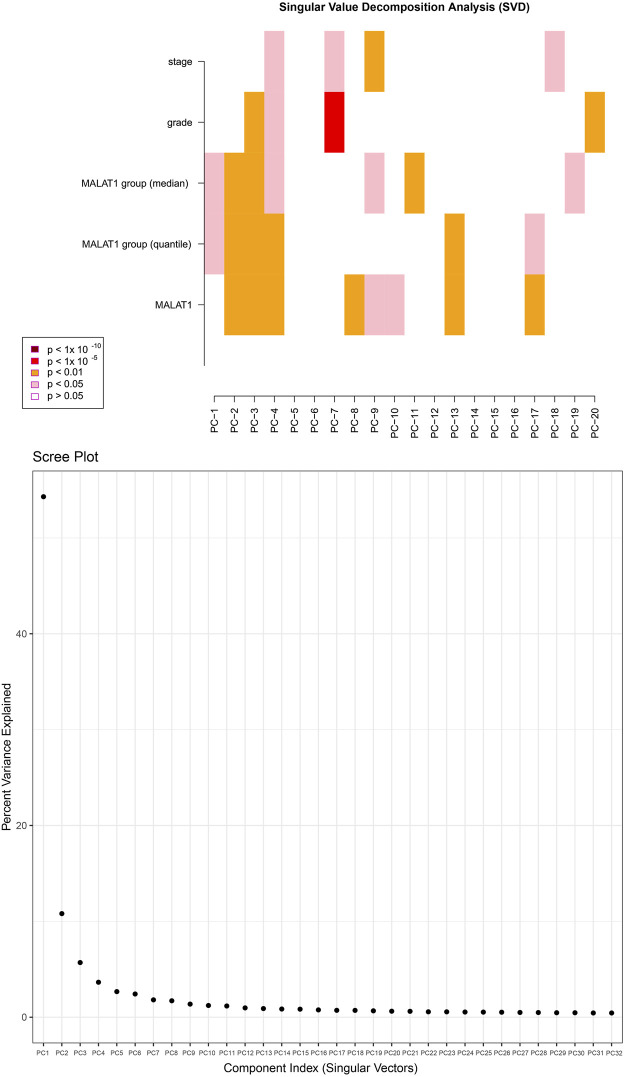
The upper plot is a heatmap of the top principal components correlating with *MALAT1*, tumor stage and tumor grade. “*MALAT1*” represents continuous variables, while “*MALAT1* group (median)” and “*MALAT1* group (quantile)” represent categorical variables grouped by median and quartiles. The color represents the degree of significance. The lower plot shows how much of the observed methylation variation was explained by the principal components.

**FIGURE 8:**
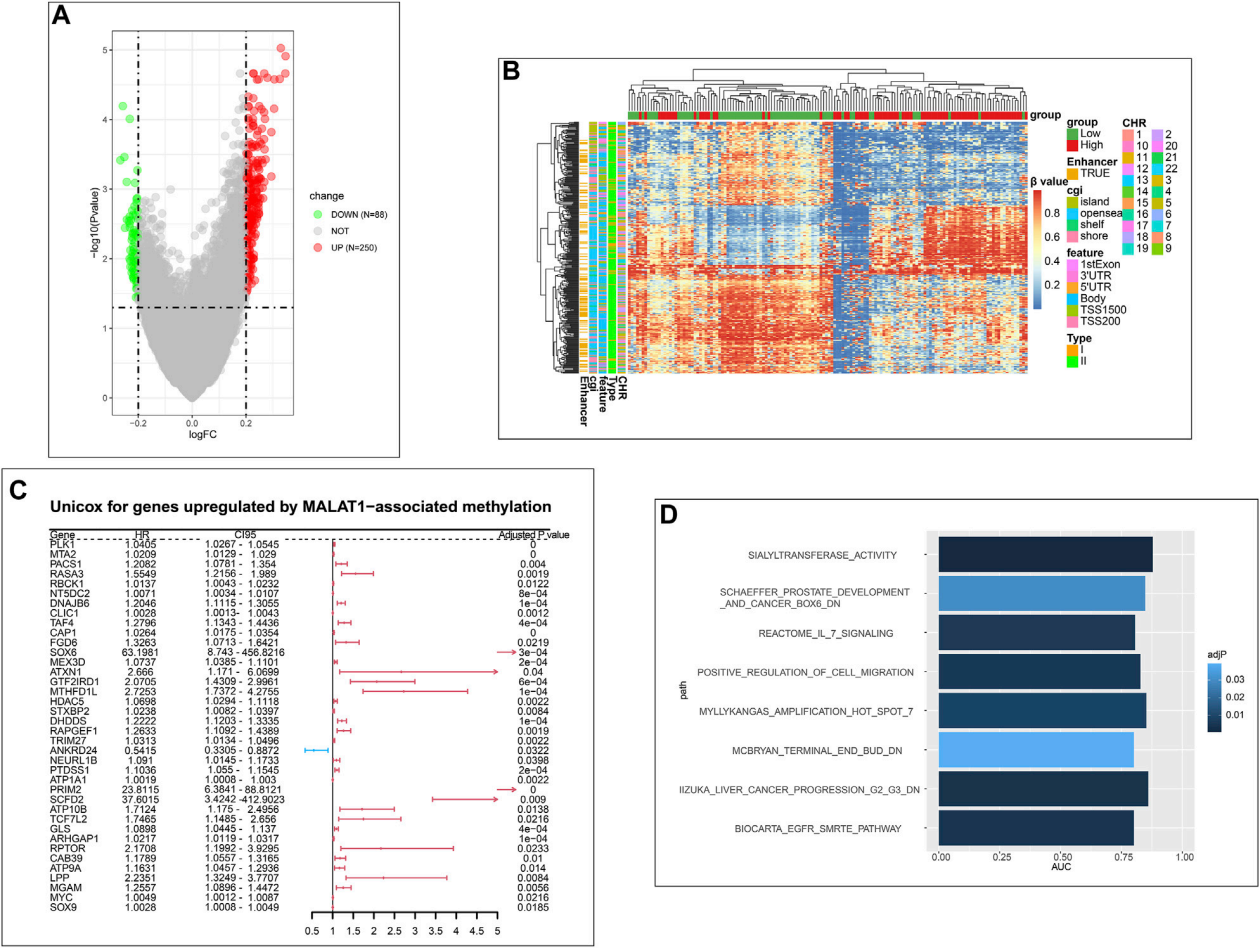
Analysis of differentially methylated positions (DMPs). **(A)** Volcano plot of DMPs. Green dots represent the positions that are hypomethylated. Red dots represent the positions that are hypermethylated. **(B)** Clustering heatmap of DMPs. Column annotations provide information about samples expressing high or low MALAT1. Row annotations provide information about DMPs. **(C)** Forest plot showing genes upregulated by MALAT1-associated methylation that were significant in Cox univariate analysis. **(D)**: Bar plot of pathways with areas under curves (AUCs) > 0.75 in the Gene Set Enrichment Analysis (GSEA).

After normalizing the methylation signal value matrix, samples expressing high or low *MALAT1* were analyzed by multidimensional scaling (Supplementary Figure S3A). The beta distributions for each sample showed that most probes were completely unmethylated (beta value ≤ 0.2), and a smaller number of probes were partially methylated (0.4 < beta value < 0.6) or completely methylated (beta value ≥ 0.8) (Supplementary Figure S3B). Sample clustering showed no outliers (Supplementary Figure S3C). The SVD plot showed that several principal components that best explained the methylation variation significantly correlated with *MALAT1* (Figure 7). More principal components significantly correlated with *MALAT1* than the clinical features of tumor stage and tumor grade, indicating that *MALAT1* correlated with methylation variants more strongly than stage or grading did (Figure 7). In samples expressing high *MALAT1,* hypomethylation occurred in 88 positions and hypermethylation in 250 positions (Figure 8A).

Beta values of DMPs were visualized using a clustering heatmap (Figure 8B). In the case of high *MALAT1* expression, 157 DMPs were hypermethylated and located in coding regions of genes, while 28 DMPs were hypomethylated and located in promoters and TSS. These DMPs may positively correlated with gene expression. Conversely, 60 DMPs were hypomethylated and located in coding regions of genes, while 93 DMPs were hypermethylated and located in promoters and TSS. These DMPs therefore may negatively correlated with gene expression (Supplementary Figure S4).

We further analyzed the correlation of these genes with *MALAT1* expression at the transcriptome level. We assumed that 87 genes were upregulated by *MALAT1* through methylation, and confirmed that their expression positively correlated with that of *MALAT1*. Conversely, three genes were assumed down-regulated by *MALAT1* through methylation, and their expression negatively were confirmed correlated with that of *MALAT1* (Supplementary Figure S5).

Univariate Cox regression was performed for 87 up-regulated genes and 3 down-regulated genes, of which 38 were significantly upregulated; all genes emerged as risk factors for poor survival except *ANKRD24,* which was a protective factor (Figure 8C). Eight terms with AUCs > 0.75 in GSEA were associated with tumors. One of the terms “IIZUKA_LIVER_CANCER_PROGRESSION_G2_G3_DN” was directly related to liver cancer progression (Figure 8D). Therefore, the *MALAT1*-related methylation probes were associated with HCC progression and survival of patients.

### *MALAT1* expression is associated with tumor signatures and TILs

**FIGURE 9:**
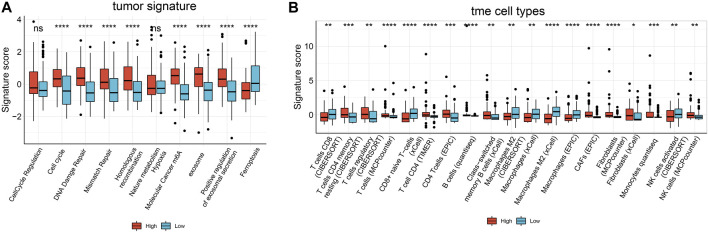
Boxplot of the results of Immuno-Oncology-Biological-Research (IOBR) analysis comparing samples expressing high or low *MALAT1* in terms of **(A)** tumor-associated signatures and **(B)** cells within the tumor microenvironment. *p < 0.05, **p < 0.01, *** < 0.001, ****p < 0.0001.

In tumor signatures and TIL analysis, we found significant enrichment in some tumor-related signatures scores in samples expressing high *MALAT1*. These signatures included genes involved in the cell cycle, DNA damage repair (DDR), mismatch repair, homologous recombination, molecular cancer m6A, exosome, and positive regulation of exosomal secretion (Figure 9). In contrast, ferroptosis scores were significantly lower in samples expressing high *MALAT1*.

We used six algorithms to estimate TILs in HCC. Several algorithms showed consistent results for the analysis of macrophages and fibroblasts. Macrophage scores were significantly higher in samples expressing low *MALAT1*, whereas fibroblasts scores were significantly higher in samples expressing high *MALAT1* (*p* < 0.05; Figure 9). In analyses of other cell types, there were few significant results or different algorithms were inconsistent in predicting the trends (Figure 9).

### *MALAT1* is associated with HCC progression

**FIGURE 10:**
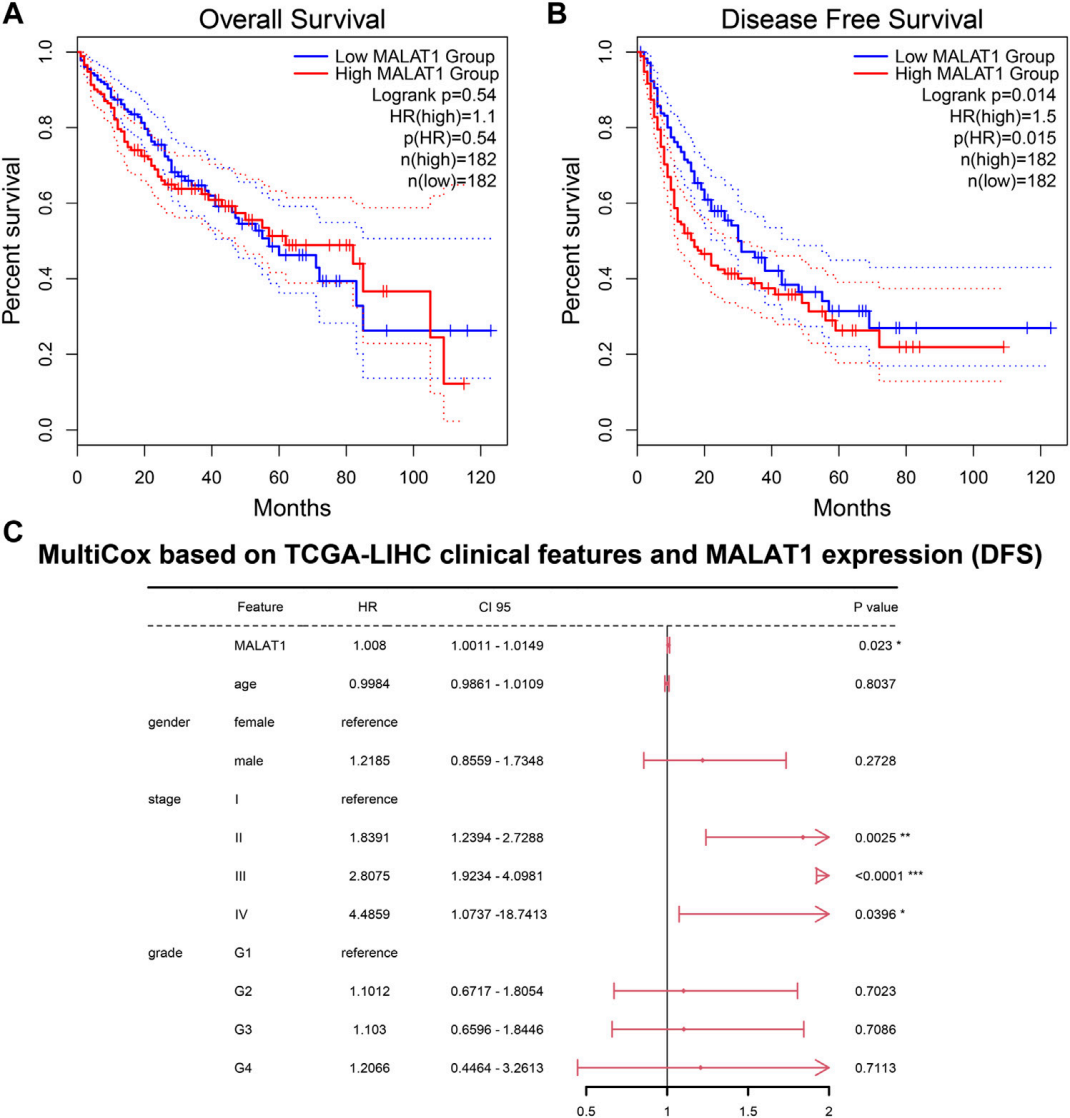
Prognosis analysis based on the TCGA LIHC cohort. **(A)** Overall survival and **(B)** Disease-free survival. Survival curves of patients with MALAT1 high expression or low expression were compared using the log-rank test. **(C)** A multivariate Cox regression model for disease-free survival containing MALAT1 and baseline clinical information. *p < 0.05; **p < 0.01; ***p < 0.001.

Among HCC patients in the TCGA-LIHC cohort, there was no significant difference in OS between those with high or low *MALAT1* expression (Figure 10A). PFS was significantly shorter in patients with high *MALAT1* expression than in those with low expression (Figure 10B; *p* = 0.014). In a multivariate Cox regression model for *MALAT1* expression and baseline clinical information (age, gender, tumor stage, and tumor grade), *MALAT1* independently predicted shorter PFS (Figure 10C; *p* = 0.023).

### General characteristics of study subjects

The median age of the 179 HCC patients was 51 years (19–77 years). There were 160 males and 19 females. The median diameter of tumors was 5.5 cm (range, 2–19 cm). The proportion of patients positive for HBsAg was 86.6%, and the proportion with serum AFP was 65.4%. The baseline features of the cases are shown in Table 1.

**TABLE 1:** Association between *MALAT1* expression and clinicopathological variables in 179 HCC patients.

Variable	n	Relative *MALAT1* expression	|Z/F|	*P*
		Median (Q_L_-Q_U_)		
Age (years)				
≥50	95	15.86 (5.12–55.29)	0.893	0.372
<50	84	18.50 (7.08–52.25)		
Sex				
Male	160	18.00 (6.55–51.53)	0.782	0.434
Female	19	9.51 (3.34–60.36)		
Family history of HCC				
yes	48	16.12 (5.74–65.23)	0.147	0.884
no	131	17.40 (6.07–49.58)		
Alcohol consumption				
Yes	56	18.50 (6.80–58.95)	0.495	0.621
No	123	16.14 (5.83–49.58)		
Liver cirrhosis				
Yes	130	23.79 (7.03–60.27)	2.701	0.007*
No	49	12.27 (3.27–31.13)		
HBsAg				
(+)	154	18.00 (6.70–56.52)	2.093	0.036*
(−)	25	9.11 (3.02–31.13)		
AFP				
(+)	115	24.03 (6.77–60.36)	2.068	0.039*
(−)	64	13.70 (3.73–45.08)		
ALT (µ/L)				
<1 ULN	92	15.35 (5.85–53.32)	0.185	0.911
1–2 ULN	65	16.23 (6.34–57.00)		
>2 ULN	22	21.93 (7.43–41.14)		
AST (µ/L)				
<1 ULN	91	15.36 (5.61–54.04)	1.925	0.382
1–2 ULN	67	22.58 (9.31–59.23)		
>2 ULN	21	11.97 (3.96–41.89)		
TBIL (μmol/L)				
<21	152	15.15 (5.74–51.53)	1.761	0.078
≥21	27	31.13 (15.27–66.53)		
PT (Sec)^a^				
<11	18	33.57 (21.89–67.92)	5.980	0.05
11–16	156	14.87 (5.62–47.45)		
≥17	3	23.55 (13.75–80.10)		
ALB (g/L)				
<35	21	28.94 (3.96–54.52)	0.211	0.833
≥35	158	16.19 (6.13–54.76)		
Tumor number (tumor nodes)				
1	146	16.91 (5.8–52.24)	0.517	0.605
≥2	33	16.14 (8.70–68.61)		
Vaso-invasion (vascular invasion)				
Yes	59	45.82 (8.65–98.93)	3.82	<0.001*
No	120	14.87 (5.81–36.42)		
Tumor capsular infiltration				
No capsule or infiltration	53	44.84 (5.77–86.76)	2.205	0.027*
Complete capsule	126	15.46 (6.06–41.38)		
Child-Pugh classification^a^				
A	163	15.86 (5.95–51.89)	0.647	0.518
B	14	27.88 (9.18–52.89)		
Tumor diameter (size, cm)				
≤5	79	16.39 (4.54–56.84)	0.084	0.933
>5	100	16.33 (6.97–45.99)		
Clinical TNM stage				
I	137	16.42 (5.60–51.17)	0.773	0.440
II–III	42	15.75 (8.20–68.82)		

^a^ 2 cases absent.

*p < 0.05. AFP, alpha-fetoprotein; ALB, albumin; ALT, alanine aminotransferase; AST, aspartate aminotransferase; HBsAg, hepatitis B surface antigen; HCC, hepatocellular carcinoma; MALAT1, metastasis-associated lung adenocarcinoma transcript 1; PT, prothrombin time; Q_L_-Q_U_, quartile range; TBIL, total bilirubin.

### *MALAT1* expression in HCC tumors and PANTs

**FIGURE 11:**
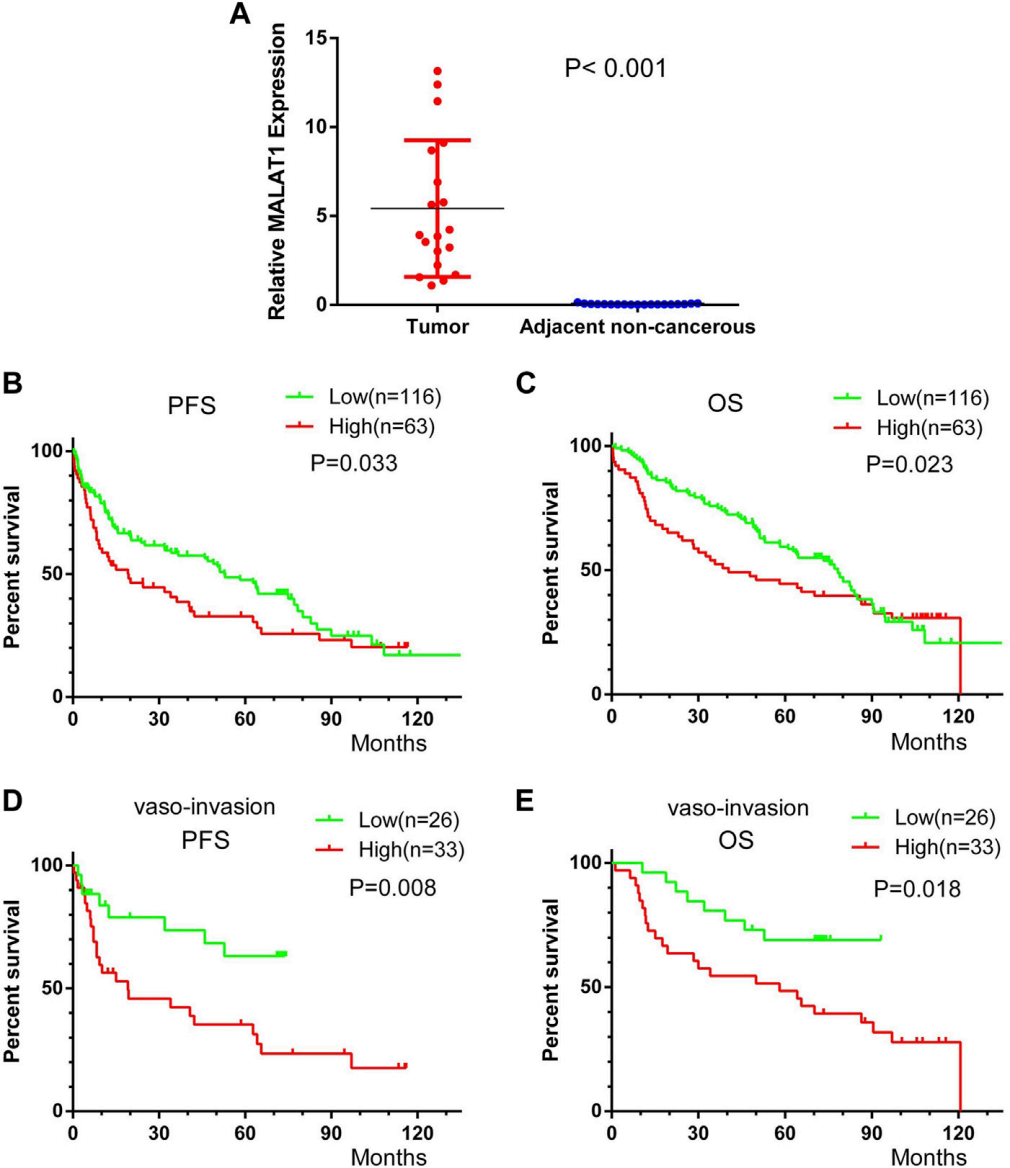
Kaplan-Meier survival curves of HCC patients after radical resection, stratified by low or high *MALAT1* expression. **(A)** MALAT1 expression in 19 paired HCC and adjacent non-cancerous tissues from patients who underwent curative resection. **(B,C)** Progression-free survival (PFS) and overall survival (OS) of all patients. **(D,E)** PFS and OS of 59 patients with vascular invasion. Curves were compared using the log-rank test.

Median relative expression of *MALAT1* was significantly higher in cancer tissues than in PANTs (5.42 ± 3.84 vs. 0.048 ± 0.032, *p* < 0.001), confirming that *MALAT1* was markedly upregulated in HCC (Figure 11A).

### Correlation between *MALAT1* expression and clinicopathological features of HCC patients

Subgroup analysis showed that patients with a history of liver cirrhosis, patients who were positive for HBsAg, patients who were positive for AFP, patients with vascular invasion and patients with tumor capsule infiltration had significantly higher *MALAT1* expression than the corresponding patient subgroups without these characteristics (all *p* < 0.05). Nevertheless, there was no significant correlation between *MALAT1* expression and age, sex, tumor diameter or number, Child-Pugh classification, clinical TNM stage, serum level of transaminase, total bilirubin, or albumin (all *p* ≥ 0.05; Table 1).

### Correlation between *MALAT1* expression and prognosis of HCC patients

Across all 179 patients, the median follow-up duration was 41 months (range, 23–59 months) for PFS and 70 months (range, 55–85 months) for OS. Patients were divided based on the mean value of *MALAT1* relative expression level of 38.76 into a high expression group (*n* = 63) and low expression group (*n* = 116).

Patients with high *MALAT1* expression were with 19.2 months mPFS and 40.5 months mOS and patients with low *MALAT1* expression were with 52.8 months mPFS and 78.3 months mOS. Patients with high *MALAT1* expression showed significantly shorter PFS than those with a low *MALAT1* expression (*p* = 0.033, Figure 11B). Similarly, they showed significantly shorter OS (*p* = 0.023, Figure 11C).

In 59 patients with vascular invasion, the mPFS of patients with *MALAT1* overexpression and low expression were 14 months and 49 months, and PFS was significantly shorter among those with high *MALAT1* expression than among those with low expression (HR 2.63, 95% CI 1.294–5.346; *p* = 0.008, Figure 11D). Similarly, the mOS of patients with *MALAT1* overexpression and low expression were 58 and 72 months, and OS was significantly shorter among those with high *MALAT1* expression (HR 2.416, 95% CI 1.160–5.031; *p* = 0.018, Figure 11E).

*MALAT1* overexpression was associated with shorter PFS and OS in subgroups who were positive for HBsAg or AFP (Table 2). Among 130 patients with liver cirrhosis, high *MALAT1* expression was associated with significantly shorter OS than low expression (*p* = 0.018) and with a tendency toward shorter PFS (*p* = 0.0566; Table 2). In contrast, the expression level of *MALAT1* was not significantly associated with PFS or OS among patients without vascular invasion, without cirrhosis, who were negative for HBsAg or who were negative for AFP (Table 2).

**TABLE 2:** Univariate analysis to identify factors associated with PFS or OS in different subgroups of HCC patients.

Clinical variable	n	*MALAT1* expression		PFS		OS	
		Low	High	|F|	P	|F|	P
Vaso-invasion							
Yes	59	26 (44.07%)	33 (55.93%)	5.623	0.0177*	5.797	0.0161*
No	120	90 (75%)	30 (25%)	2.621	0.1055	2.772	0.0959
Tumor capsular infiltration							
No capsule or infiltration	53	25 (47.17%)	28 (52.83%)	2.236	0.1348	3.005	0.0830
Complete capsule	126	91 (72.22%)	35 (27.78%)	3.515	0.0608	2.476	0.1156
HBsAg							
(+)	154	96 (62.34%)	58 (37.66%)	4.230	0.0397*	5.675	0.0172*
(−)	25	20 (80%)	5 (20%)	0.166	0.6834	0.108	0.7425
AFP							
(+)	115	71 (61.74%)	44 (38.26%)	4.249	0.0393*	3.875	0.0490*
(−)	64	45 (70.31%)	19 (29.69%)	1.814	0.1780	2.173	0.1405
Liver cirrhosis							
Yes	130	76 (58.46%)	54 (41.54%)	3.635	0.0566	5.597	0.0180*
No	49	40 (81.63%)	9 (18.37%)	3.729	0.0535	0.750	0.2865

Test, Gehan-Breslow-Wilcoxon test. |F|, Effect size generated in the Gehan-Breslow-Wilcoxon test, which showed a chi-squared distribution. *p < 0.05. AFP, alpha-fetoprotein; HBsAg, hepatitis B surface antigen.

### Prognostic factors in patients with HCC after hepatectomy

Univariate analysis showed that sex, HBsAg, tumor number, tumor diameter, TNM stage, and *MALAT1* expression were significantly associated with OS and PFS (Table 3).

**TABLE 3:** Comparison of PFS and OS between HCC patients expressing low or high *MALAT1* levels, stratified by clinicodemographic variables.

Variable	*MALAT1* expression		PFS		OS	
	Low	High	|F|	P	|F|	P
Age (years)						
≥50	64 (67.37%)	31 (32.63%)	2.497	0.114	0.887	0.346
<50	52 (61.9%)	32 (38.1%)				
Sex						
Male	104 (65%)	56 (35%)	4.908	0.027*	5.953	0.015*
Female	12 (63.16%)	7 (36.84%)				
Family history of HCC						
Yes	31 (64.58%)	17 (35.42%)	0.831	0.362	0.594	0.441
No	85 (64.89%)	46 (35.11%)				
Alcohol consumption						
Yes	34 (60.71%)	22 (39.29%)	0.073	0.788	0.055	0.815
No	82 (66.67%)	41 (33.33%)				
Liver cirrhosis						
Yes	76 (58.46%)	54 (41.54%)	0.701	0.402	0.981	0.321
No	40 (81.63%)	9 (18.37%)				
HBsAg						
(+)	96 (62.34%)	58 (37.66%)	7.714	0.005*	6.435	0.011*
(−)	20 (80%)	5 (20%)				
AFP						
(+)	71 (61.74%)	44 (38.26%)	2.452	0.117	0.926	0.336
(−)	45 (70.31%)	19 (29.69%)				
Tumor number						
Single	96 (65.75%)	50 (34.25%)	8.429	0.004*	8.057	0.005*
≥2	20 (60.61%)	13 (39.39%)				
Vaso-invasion						
Yes	26 (44.07%)	33 (55.93%)	0.573	0.449	0.530	0.466
No	90 (75%)	30 (25%)				
Tumor capsular infiltration						
Yes	25 (47.17%)	28 (52.83%)	0.000	1.00	0.089	0.766
No	91 (72.22%)	35 (27.78%)				
Child-Pugh classification						
A	107 (65.64%)	56 (34.36%)	0.126	0.723	0.064	0.801
B	9 (64.29%)	5 (35.71%)				
Tumor size (cm)						
≤5	49 (62.03%)	30 (37.97%)	4.654	0.031*	3.940	0.047*
>5	67 (67%)	33 (33%)				
Clinical TNM stage						
I	90 (65.69%)	47 (34.31%)	6.132	0.013*	5.110	0.024*
II–III	26 (61.9%)	16 (38.1%)				

Test, Gehan-Breslow-Wilcoxon test. |F|, effect size from the Gehan-Breslow-Wilcoxon test, which showed a chi-squared distribution. *p < 0.05. AFP, alpha-fetoprotein; HBsAg, hepatitis B surface antigen.

**FIGURE 12:**
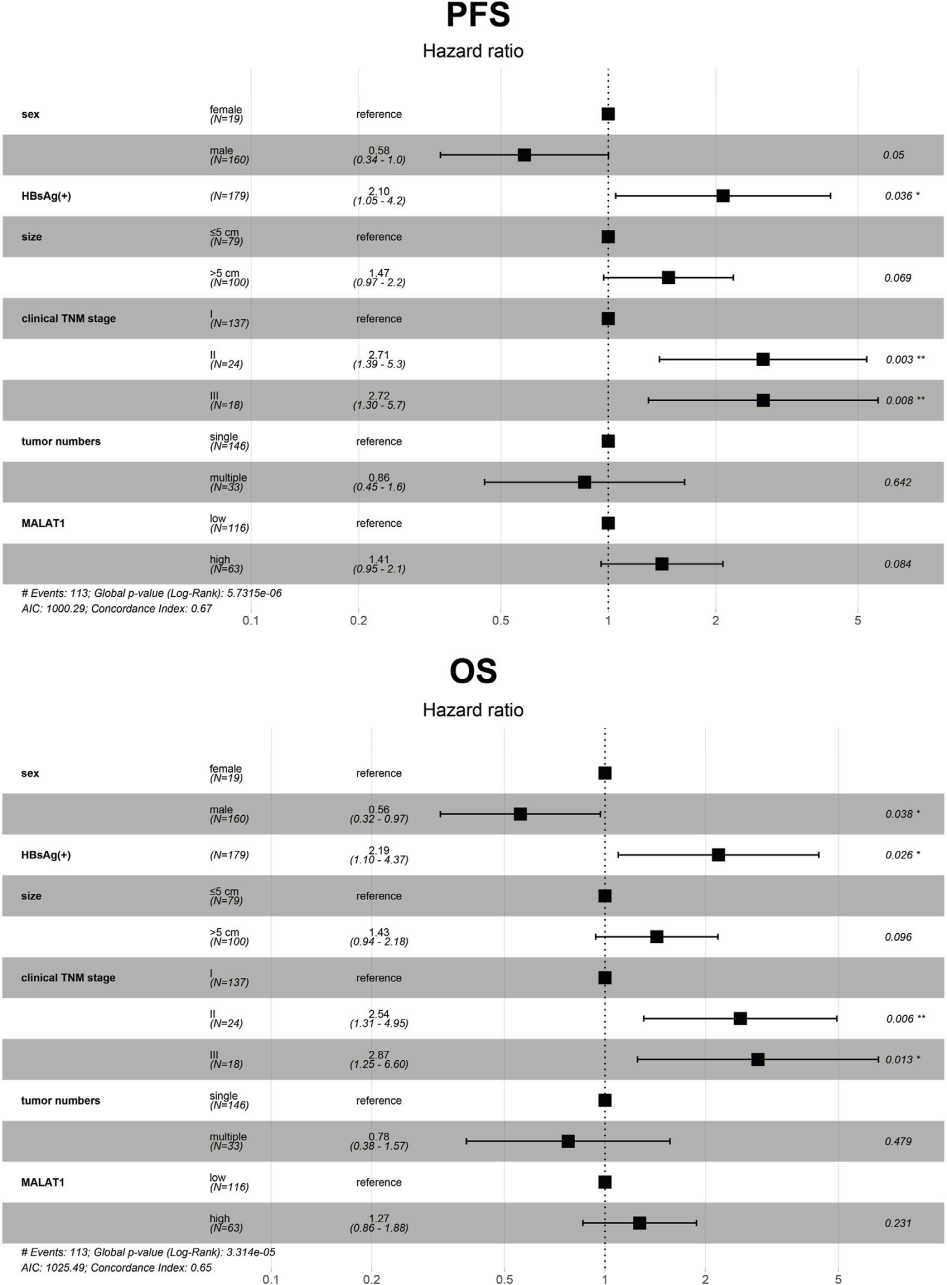
Multivariate analysis of 179 HCC patients to identify independent risk factors of progression-free survival and overall survival. The dots and bars represent the HR and 95% CI, respectively. CI, confidence interval; HR, hazard ratio; *MALAT1*, metastasis-associated lung adenocarcinoma transcript 1; OS, overall survival; PFS, progression-free survival.

Variables with *p* < 0.05 in the univariate analysis were included in the multivariate Cox analysis. Furthermore, the results showed that the independent risk factors for OS included sex, HBsAg, and clinical TNM stage (*p* < 0.05; Figure 12). At the same time, HBsAg and clinical TNM stage were independent risk factors for PFS (*p* < 0.05; Figure 12). The *p*-values of tumor size from OS and PFS multivariate Cox analysis was less than 0.1, and the *p*-value of *MALAT1* expression from PFS multivariate Cox analysis was less than 0.1 (Figure 12).

### A prognostic model based on *MALAT1* in combination with other prognostic indicators has moderate predictive power

**FIGURE 13:**
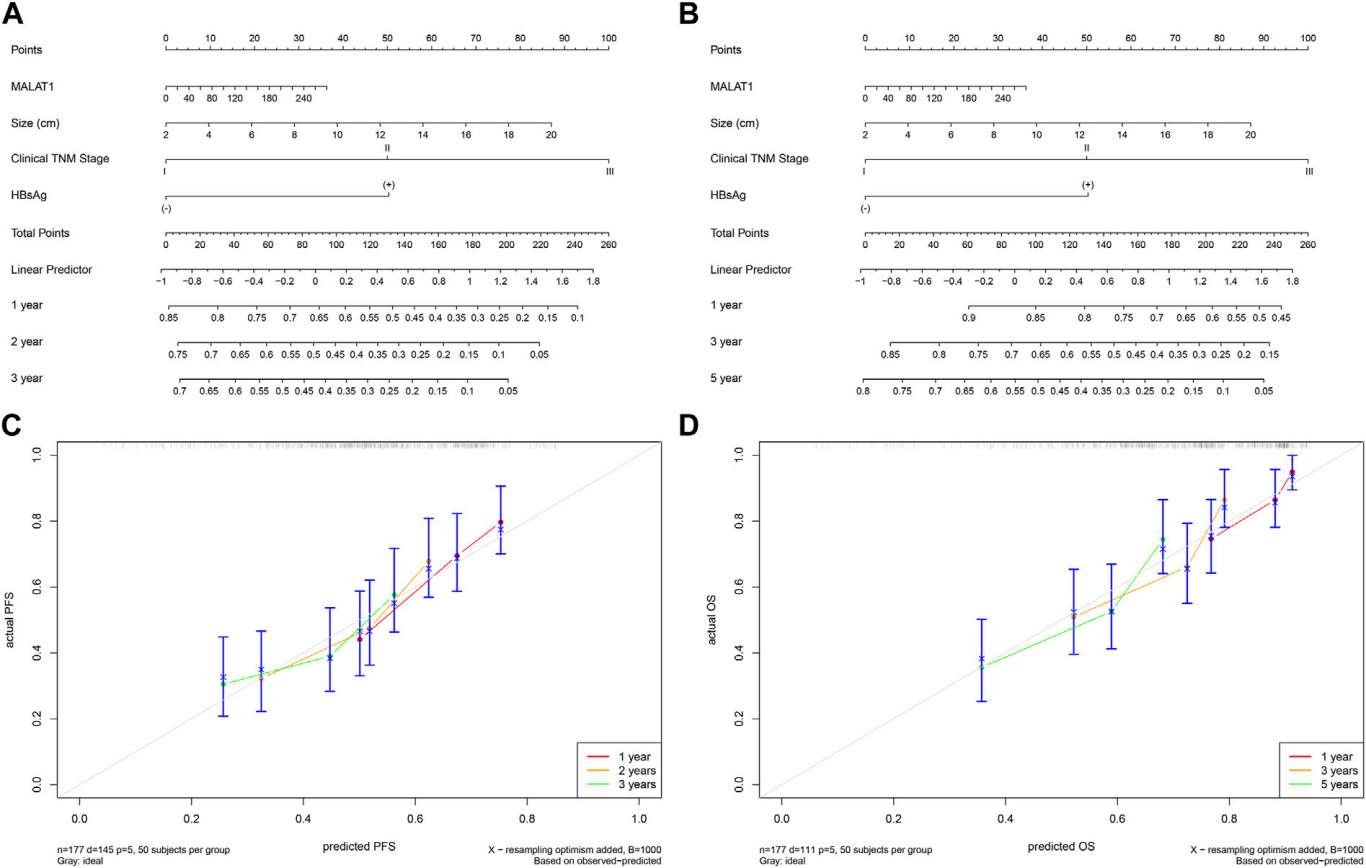
Progression and survival prediction models. **(A)**: Nomogram of the progression prediction model. **(B)**: Nomogram of the survival prediction model. **(C)**: Calibration curve for the progression prediction model. **(D)**: Calibration curve for the survival prediction model.

**FIGURE 14:**
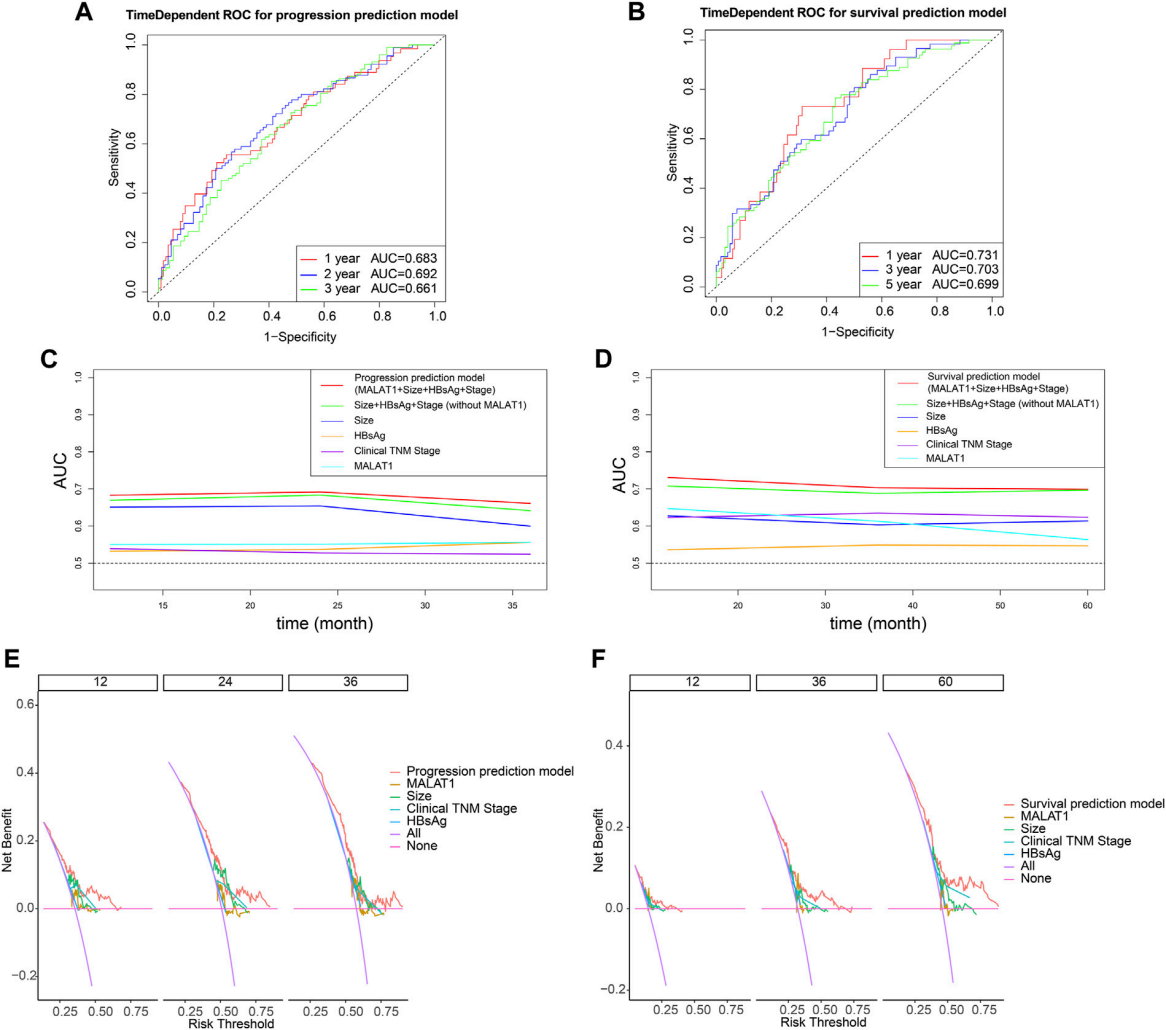
Evaluation of model performance using time-dependent receiver operating characteristic curves. **(A)** Predictive power of the progression prediction model. **(B)** Predictive power of the survival prediction model. **(C)** Comparisons of areas under curves (AUCs) for progression prediction model, other prognostic parameters and MALAT1 alone. **(D)** Comparisons of areas under curves (AUCs) for survival prediction model, other prognostic parameters and MALAT1 alone. **(E)**: Decision curves for the progression prediction model, other prognostic parameters and MALAT1 alone. **(F)**: Decision curves for the survival prediction model, other prognostic parameters and MALAT1 alone.

The multivariate Cox analysis showed that there was strong collinearity between the variable “tumor number” and “clinical TNM stage” and the prognostic value of “sex” was not clear. Therefore, we constructed prediction models based on four promising variables: “*MALAT1*,” “size,” “clinical TNM stage,” and “HBsAg” (Figures 13A, B). The calibration curves showed that the progression prediction model fit the data well at 1, 2 and 3 years (Figure 13C), while the survival prediction model fit the data moderately well at 1, 3, and 5 years (Figure 13D). Using the progression prediction model, we obtained AUCs of 0.683 for 1-year progression rate, 0.692 for 2-year progression rate, and 0.661 for 3-year progression rate (Figure 14A). Using the survival prediction model, we obtained AUCs of 0.731 for 1-year survival rate, 0.703 for 3-year survival rate, and 0.699 for 5-year survival rate (Figure 14B). Time-dependent AUCs showed that the prediction models outperformed the predictions of individual indicators. Predictive models constructed with other indicators excluding *MALAT1* were slightly inferior to *MALAT1*-based predictive models, for both PFS and OS. The performance of *MALAT1* alone was not inferior to that of clinical stage and HBsAg in the prediction of progression, or from that of tumor size and HBsAg in the prediction of survival when data were censored at > 40 months (Figures 14C, D). Decision curves were plotted by depicting the net benefit ratio along the vertical axis and the risk threshold along the horizontal axis (Figures 14E, F). The results showed that the smaller the risk threshold value, the greater the net benefit. Over most threshold intervals, the progression and survival prediction models led to greater net benefit than models based on other independent indicators.

### Diagnostic value of *MALAT1* for clinicopathological features

**FIGURE 15:**
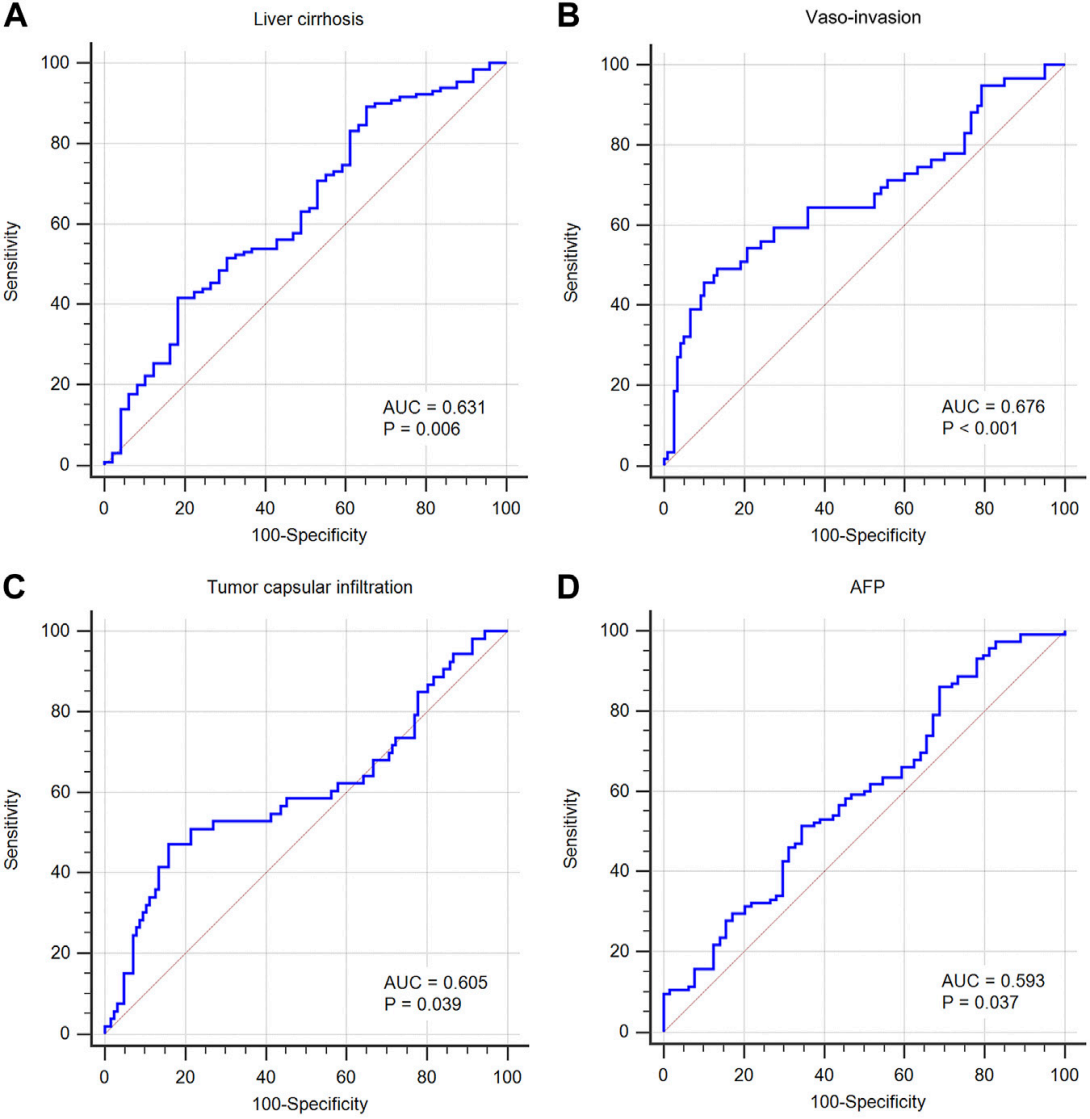
Predictive value of *MALAT1* for diagnosing clinicopathological characteristics, based on the area under the time-dependent receiver operating characteristic curve (AUC). **(A)** AUC was 0.631 for cirrhosis (95% CI: 55.6%–70.2%, *p* = 0.006). **(B)** AUC was 0.676 for vascular invasion (95% CI: 60.2%–74.4%, *p* < 0.001). **(C)** AUC was 0.605 for tumor capsular infiltration (95% CI: 52.9%–67.7%, *p* = 0.039). **(D)** AUC was 0.593 for AFP positive (95% CI: 51.8%–66.6%, *p* = 0.037).

Using an appropriate cut-off value, the AUC of *MALAT1* expression level used to distinguish patients with or without liver cirrhosis was 0.631 (cut-off value 3.86, *p* = 0.006; Table 4; Figure 15A), 0.676 for patients with or without vascular invasion (cut-off value 54.52, *p* < 0.001; Figure 15B), 0.605 for patients with or without tumor capsule infiltration (cut-off value 54.52, *p* = 0.039; Figure 15C), and 0.593 for patients positive or negative for AFP (cut-off value 4.23, *p* = 0.037; Figure 15D).

**TABLE 4:** Assessment of the ability of *MALAT1* to diagnose clinicopathological characteristics in HCC patients undergoing radical resection.

Characteristic	Cut-off *MALAT1* expression level	Se (%)	Sp (%)	AUC	P	HR 95% *CI*	
						Low	High
Liver cirrhosis	3.86	89.2	34.7	0.631	0.006	0.556	0.702
Vaso-invasion	54.52	49.2	86.7	0.676	<0.001	0.602	0.744
Tumor capsular infiltration	54.52	47.2	84.1	0.605	0.039	0.529	0.677
AFP	4.23	86.1	31.2	0.593	0.037	0.518	0.666

AFP, alpha-fetoprotein; AUC, area under the receiver operating characteristic curve; CI, confidence interval; HR, hazard ratio; Se: sensitivity; Sp: specificity.

## Discussion

*MALAT1* was initially reported as a biomarker of metastasis in the early lung adenocarcinoma (21). Increasingly investigations showed that *MALAT1* was not only a reliable metastasis-related biomarker but also a vital regulator involved in occurrence, invasiveness, drug resistance, and metastasis of various cancers such as HCC and lung cancer. Studies have revealed that *MALAT1* overexpression is significantly related to patient outcomes. For example, upregulated *MALAT1* is associated with poor disease-free survival and OS in patients with middle thoracic esophageal squamous cell carcinoma (22) and osteosarcoma (23). Recently, several studies have been conducted to investigate the prognostic value of *MALAT1* in HCC, but the results have been inconsistent. Lai et al. (24) found that *MALAT1* was overexpressed in 9 liver cancer cell lines and 112 HCC tissues, and survival analysis showed that patients with high level of *MALAT1* had a significantly increased risk of recurrence after liver transplantation. Multivariate analysis confirmed that *MALAT1* was an independent prognostic factor for predicting the recurrence of HCC (HR 3.280, *p* = 0.003). However, another study obtained quite different results: Sonohara et al. found increased expression of *MALAT1* in HCC and that *MALAT1* expression in HCC was associated with better survival (25). Therefore, the prognostic value of *MALAT1* in HCC needs to be investigated by a broader range of research methods.

Since *MALAT1* is a lncRNA, it is not directly involved in protein coding but instead it regulates the expression of coding genes through mechanisms such as ceRNA (14) and perhaps also methylation and mutation (15, 26). In this study, we investigated the relationships of *MALAT1* with protein-coding genes, ceRNAs, methylation profiles, and mutation profiles, thus identifying various ways in which *MALAT1* affects the survival of HCC patients. First, we used text mining to find *MALAT1*-interacting proteins, half of whose coding genes were significantly associated with the poor survival of HCC. Enrichment analysis suggested a high correlation between *MALAT1*-interacting proteins and HCC. These analyses suggest that *MALAT1*-interacting proteins may mediate the observed association of *MALAT1* with prognosis of HCC patients. The high correlation between *MALAT1*-interacting proteins and HCC also suggests that *MALAT1* has a vital role in the pathogenesis underlying HCC. Indeed, five prognosis-related *MALAT1*-interacting proteins, *AGO2, HNRNPC, EZH2, SFPQ,* and *SRSF1*, have previously been linked to hepatotumorigenesis. *AGO* promotes cell proliferation and angiogenesis in HCC (27, 28); silencing *HNRNPC* inhibits proliferation, migration, and invasion of HCC cells (29); *EZH2* enhances protein kinase B activation to promote HCC progression (30); *SFPQ* plays an important role in the enhancement of fatty acid biosynthesis by NONO to promote HCC progression (31); and *SRSF1* promotes HCC development, which can be regulated by *MALAT1* (32, 33).

The most important link between *MALAT1* and HCC pathogenesis appears to be mediated by *MALAT1* acting as a ceRNA to affect the target pathways FOXA1/CD24/Src and PI3K/Akt/mTOR*,* as well as the target genes *IAP, ZEB1,* and *FOXM1.* Through these targets, *MALAT1* promotes HCC proliferation and angiogenesis, inhibiting apoptosis, and promoting the progression of HCC (34–38). To identify more potential target genes, we constructed a network of *MALAT1*-related ceRNAs using CLIP-seq data. Interestingly, the predicted target gene *CAMK2G* has been linked to the proto-oncogene tyrosine-protein kinase Src family, which was previously confirmed by others an *MALAT1* target (34). CAMK2G is a subunit of calcium/calmodulin-dependent protein kinase II (CaMKII). The reactome database showed that CaMKII and Src participate together in several serine threonine kinase (RAF)-related pathways or complexes (39), and that both CAMK2G and Src are targets of tyrosine kinase inhibitors (40). These predicted target genes may be linked to confirmed target genes or new, yet-to-explored mechanisms.

Half of the target genes regulated by *MALAT1* as a ceRNA were significantly associated with poor survival, suggesting that *MALAT1* may influence both HCC pathogenesis and patient prognosis. Among these, *LCOR* and *PDE7A* have been shown to play roles in other types of cancer. *LCOR* activates transcription and can promote the development of breast cancer (41) and the low differentiation of cervical intraepithelial neoplasia (42). Inhibition of *PDE7A* expression inhibits cancer cell proliferation, migration and invasion in endometrial cancer, while overexpression of *PDE7A* has the opposite effects (43). The effects of *MALAT1* regulation of its target genes and the effect of those genes on HCC should be explored.

*MALAT1* expression was also associated with mutational events in our study. Lv et al. found that *MALAT1* expression could be used as a marker of *EGFR* mutation status (44). Badalamenti et al. discovered that *MALAT1* expression was associated with *c-KIT* mutation status (26). Ak et al. revealed that the *C228T* mutation in the *TERT* gene was associated *MALAT1* expression and with worse prognosis in glioblastoma patients (45). The present study identified a correlation between *MALAT1* and more than 20 mutational events in HCC by somatic mutation data analysis. Mutational events associated with high or low *MALAT1* expression may explain the poor prognosis in HCC. In addition, we found that mutations in *IRX1* and *TP53* were more likely to occur when *MALAT1* was highly expressed and were associated with worse survival. *TP53* mutation, the most well-known cause of cancer, has been associated with adverse outcomes in HCC patients (46). Mutation in *LRP1B*, which was associated with low *MALAT1* expression in HCC, also emerged as a prognostic indicator of worse survival. Therefore, *MALAT1*-associated mutations may be one of the reasons why dysregulation of *MALAT1* expression is accompanied by poor prognosis.

In addition to genetic mutation, epigenetic modification of methylation in the promoter region has been suggested as an important alternative mechanism of tumorigenesis. Methylation of promoter regions of several genes has been associated with the development of HCC (47), and methylation features detected in tissues and serum have been used as biomarkers to predict the prognosis of patients with HCC (48). In our analysis of DNA methylation profiling data, *MALAT1* expression significantly correlated with methylation variations, suggesting that *MALAT1* as lncRNA may also have been associated with the up-or down-regulation of methylation signals at many methylation positions. Subsequently, we found that *MALAT1*-associated methylation was associated with poor survival of HCC patients and was enriched for terms associated with liver cancer progression. These findings suggest that *MALAT1* may be involved in the progression and prognosis of HCC through its association with DNA methylation. Further work is needed to elucidate the molecular pathways linking *MALAT1* expression and DNA methylation.

Based on RNA sequencing data, we transformed the gene expression profile into multiple tumor-associated signatures. *MALAT1* is co-expressed with multiple signatures that can promote HCC progression. We were able to observe that high *MALAT1* expression was accompanied by high expression of cell cycle-related genes, which is consistent with the finding of several studies that *MALAT1* overexpression promotes the cell cycle and inhibits apoptosis (49–51). Similarly, high *MALAT1* expression is accompanied by high expression of DNA repair-related genes, and studies have successfully induced DNA damage by targeting *MALAT1* (52, 53). We found *MALAT1* was positively associated with exosome characteristics, and others found that exosome-mediated intercellular communication allowed the transfer of *MALAT1* as a lncRNA to play a regulatory role (54, 55). In contrast, *MALAT1* was negatively associated with ferroptosis signature, and one study reported that silencing *MALAT1* induced ferroptosis (56), reflecting the possibility that *MALAT1* may also contribute to the development of HCC by inhibiting ferroptosis. TIL prediction analysis showed that high *MALAT1* expression was associated with several immunosuppressive signatures, such as reduced macrophage infiltration and increased fibroblast infiltration. Wang et al. found that *MALAT1* could induce fibroblast activation leading to gastric cancer progression (57). Hou et al. also found that *MALAT1* was associated with macrophage differentiation in HCC. They found that *MALAT1* negatively correlated with miR-140 and that inhibition of miR-140 polarized macrophages toward the M2 subtype and away from the M1 subtype (58). Further work is needed to clarify how *MALAT1* functions in the tumor microenvironment.

We have discussed the multiple potential ways in which *MALAT1* may be involved in the progression of HCC and the poor outcomes of HCC patients. To directly confirm the relationship between *MALAT1* and the prognosis of HCC patients, we collected tissue samples from patients and conducted RT-PCR assays. The results showed that *MALAT1* was significantly upregulated in HCC tissues relative to PANTs. Moreover, *MALAT1* overexpression was closely related to the shorter PFS and OS in HCC patients after hepatectomy, especially in patients who were positive with HBsAg, who had a tumor diameter ≥ 5.0 cm, or who had tumor number ≥ 2. Thus, *MALAT1* is associated with poor outcomes in HCC. However, different from previous results (24), the present study found that *MALAT1* overexpression was not an independent risk factor for the prognosis of HCC patients after Cox multivariate analysis. The reason for this disrepancy may be that the patients in that previous study underwent liver transplantation, whereas our patients underwent radical resection, such that variation in regenerative capacity of residual liver tissue and liver function may have confounded the effects of *MALAT1* overexpression on survival.

Molecular mechanisms of signaling pathways have shown that the upregulation of *MALAT1* expression significantly promotes the malignant phenotype of HCC cells. By promoting β-catenin expression, *MALAT1* activates the canonical Wnt signaling pathway and contributes to the formation of HCC tumor sphere, as well as the increase in CD133+ and CD90+ HCC cell populations (59). *MALAT1* is involved in HCC cellular glucose metabolism: it enhances *TCF7L2* translation and activates the mTORC1-4EBP1 axis (60). Other researchers found that the interaction of *MALAT1* and miR-124 promotes HBx-induced stem cell characteristics in HepG2 cells by regulating PI3K/Akt signaling (61). By cooperating with enhancer of zeste homolog 2 (*EZH2*), *MALAT1* promotes Snail family transcriptional repressor 1 (*SNAI1*) expression by sponging miR-22 and suppressing E-cadherin expression, ultimately promoting the epithelial-mesenchymal transition in HCC (62). The miR-3064-5p plays an anti-angiogenic role by inhibiting the FOXA1/CD24/Src pathway, and *MALAT1* sponges this miRNA to weaken its suppressive effect (34). Overexpression of *MALAT1* inhibits Mir-140, leading to overexpression of vascular endothelial growth factor A (VEGF-A) and promoting angiogenesis in human umbilical vein endothelial cells (HUVECs) (58).

Through the analysis of the relationship between *MALAT1* and other HCC-associated clinical features, we also revealed that overexpression of *MALAT1* was closely associated with clinical features such as liver cirrhosis, vascular invasion, tumor capsular infiltration, AFP positivity, and HBsAg positivity. Further analysis of the AUC curve confirmed that *MALAT1* expression was helpful in the differentiation of patients with cirrhosis, vascular invasion, capsule infiltration, and AFP positivity.

In China, chronic infection with hepatitis B virus (HBV) is one of the key risk factors for HCC, and 80% of HCC cases are complicated with liver cirrhosis caused by hepatitis (63). Yuan et al. found that *MALAT1* expression level was progressively unregulated as HCC advanced from normal liver to dysplastic liver to cirrhotic liver and finally to HCC (64). These results demonstrate an association between *MALAT1* and HCC occurrence. In addition, *MALAT1* overexpression induced proliferation and metastasis of cancer cells associated with vascular and capsular invasion in HCC (34, 58–62). The overexpression of *MALAT1* was significantly correlated with serum indicators (65). Unlike previous research (65), we found here that the expression of *MALAT1* did not significantly correlate with liver function indicators, such as alanine aminotransferase (ALT), aspartate aminotransferase (AST), or total bilirubin. The reason might be related to the differences in the types of the samples analyzed (e.g., tissue or plasma), and the clinicopathological features of the patients enrolled (e.g., type of viral hepatitis, presence or absence of liver cirrhosis). Since *MALAT1* did not have covariance with significant prognostic factors including size of tumor, number of tumors, tumor stage, and gender, we constructed prediction models for progression rate and survival rate based on *MALAT1* and those factors. The prognostic models showed moderate performance in predicting progression and survival, and the models outperformed individual indicators. These models may be a new method in the clinic to predict the outcome of HCC patients.

The main limitation of this study was also its strength: we investigated aspects of interacting proteins, ceRNAs, somatic mutations, methylation, and tumor-related features based on multi-omics data. While our analyses had a broad reach, they were difficult to integrate together in order to identify deep relationships. Future research should focus on the more promising findings to follow up in mechanistic studies. Secondly, there were some discrepancies between the results of the survival analysis based on TCGA-LIHC in this study and the analysis based on the samples we collected. These could be due to the fact that the patients in our collected sample underwent radical resection, whereas those in the TCGA-LIHC may have undergone a variety of radical or partial surgeries. The two cohorts may also have differed in additional treatments. The discrepancies may also be due to ethnic, environmental, and socioeconomic differences.

## Conclusion

*MALAT1* expression was significantly higher in hepatocellular carcinoma, and patients with a high expression level had a worse prognosis than those with a low expression level. *MALAT1* could serve as a prognostic marker for patients with hepatocellular carcinoma who have undergone liver resection.

## Data Availability

The raw data supporting the conclusion of this article will be made available by the authors, without undue reservation.
