# Supplementary figures and images for: Prognostic value of long non-coding RNA MALAT1 in hepatocellular carcinoma: A study based on multi-omics analysis and RT-PCR validation
Source: Pathol Oncol Res. 2023 Jan 4;28:1610808. doi: 10.3389/pore.2022.1610808 (PMC9845286; doi:10.3389/pore.2022.1610808)

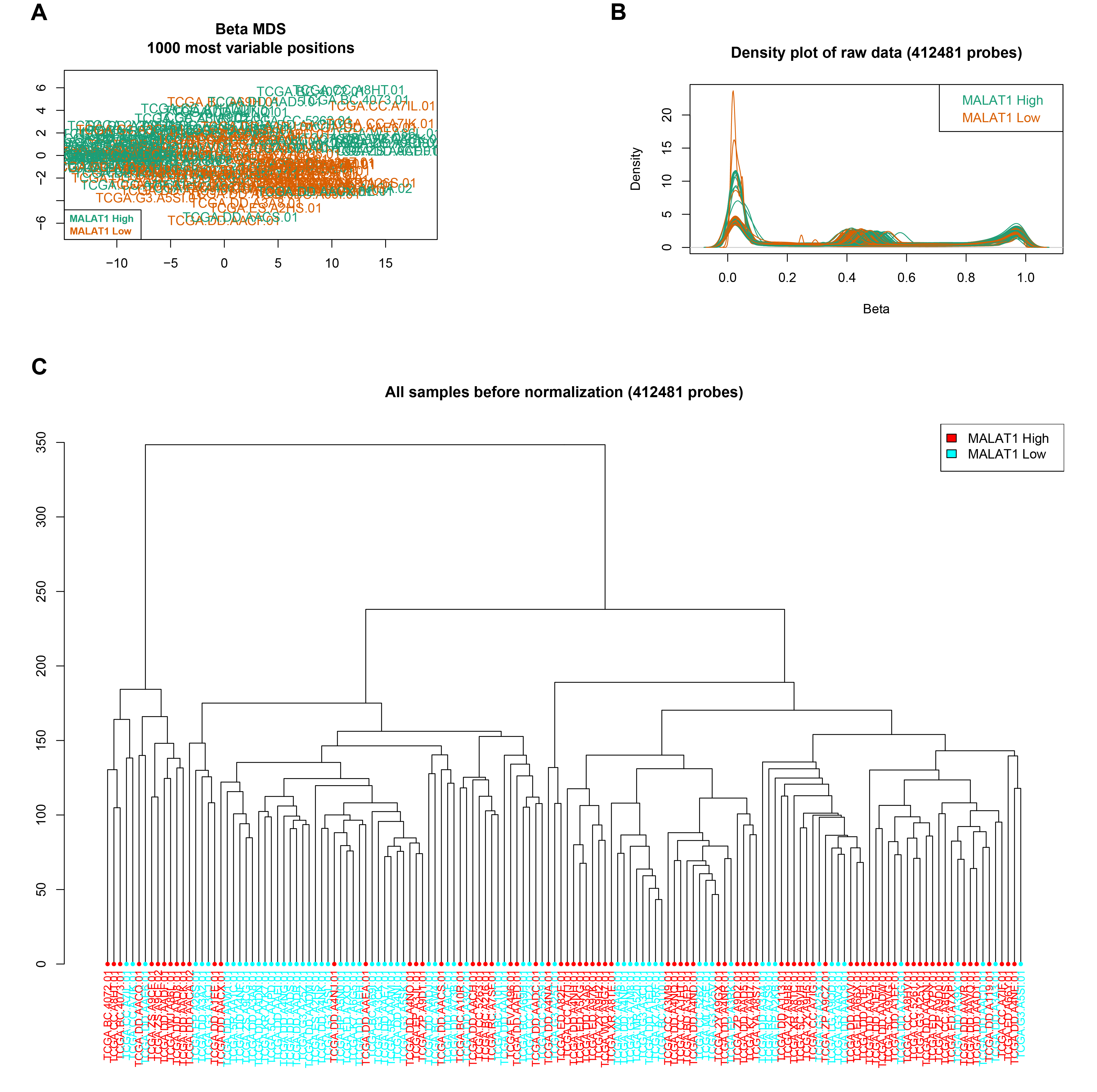

Supplement: Supplementary file 1 [file Image3.JPEG]

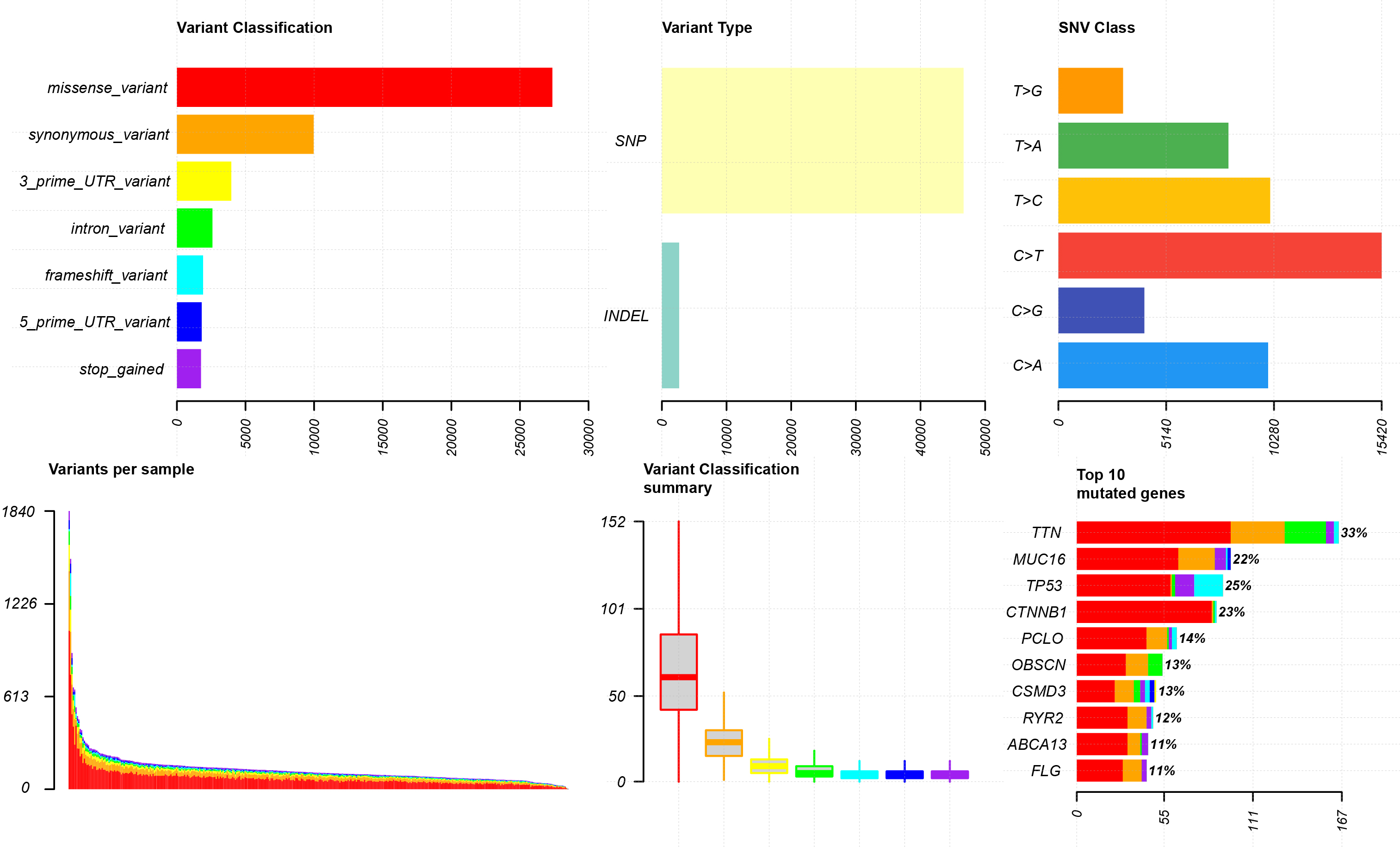

Supplement: Supplementary file 2 [file Image1.JPEG]

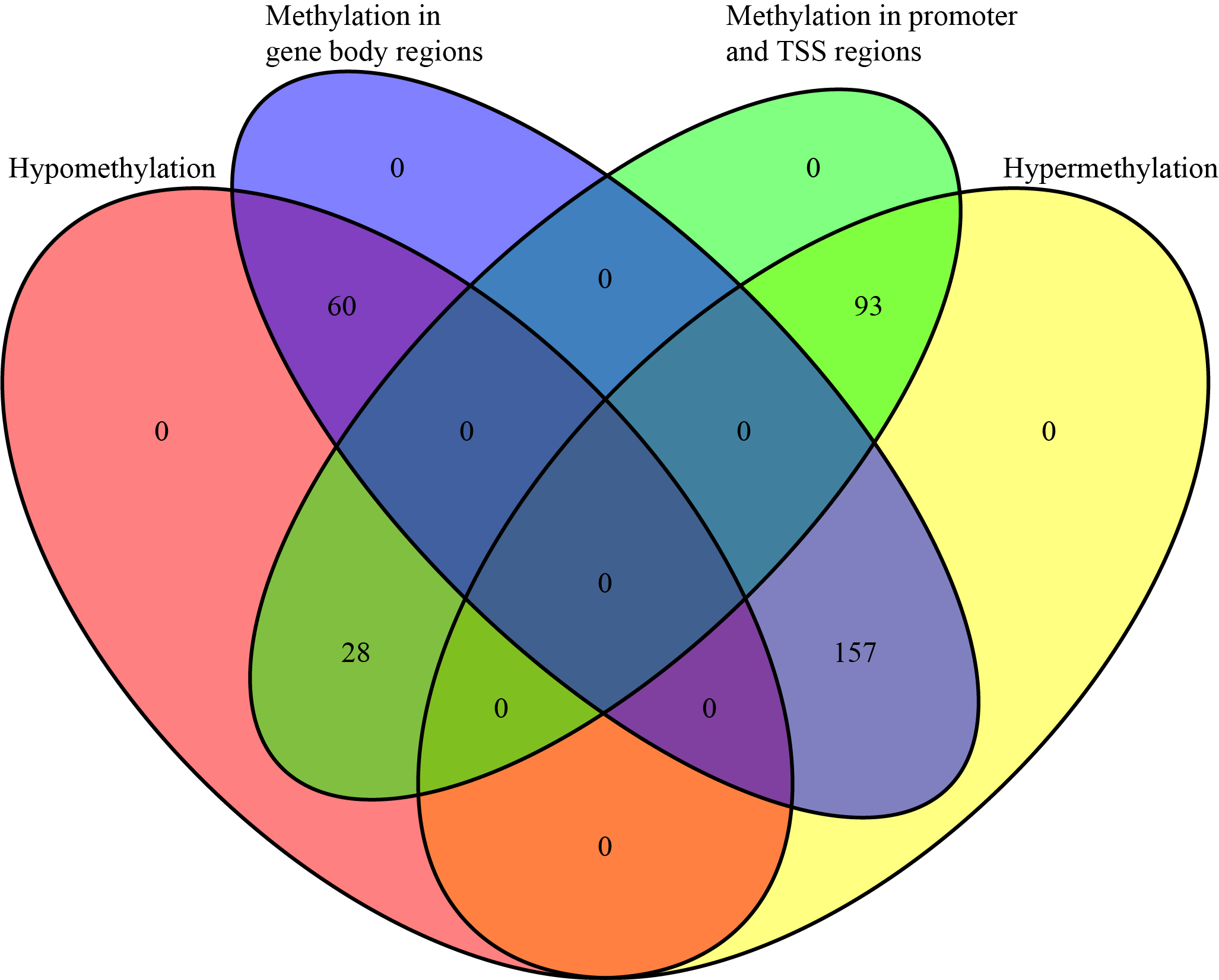

Supplement: Supplementary file 3 [file Image4.JPEG]

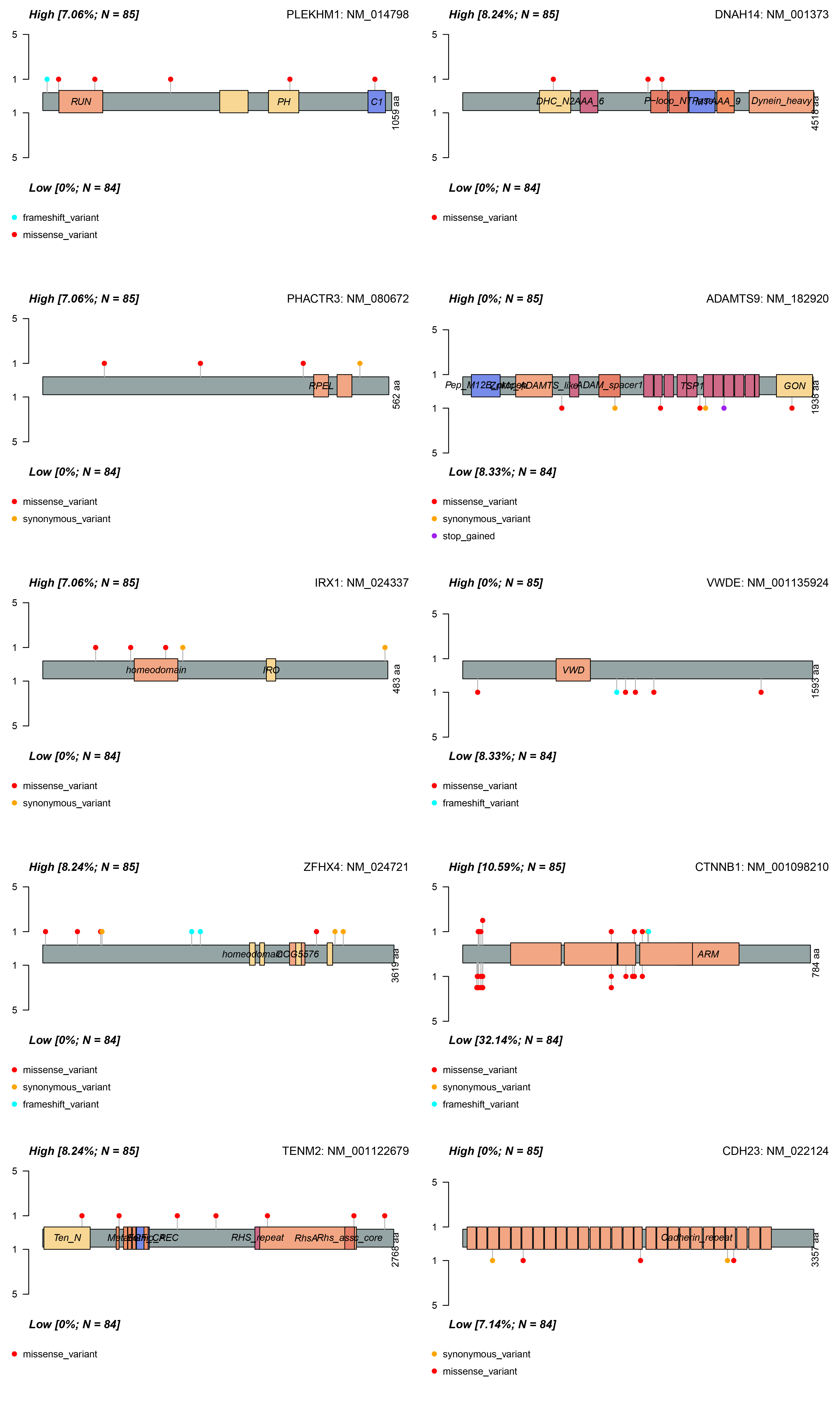

Supplement: Supplementary file 4 [file Image2.JPEG]

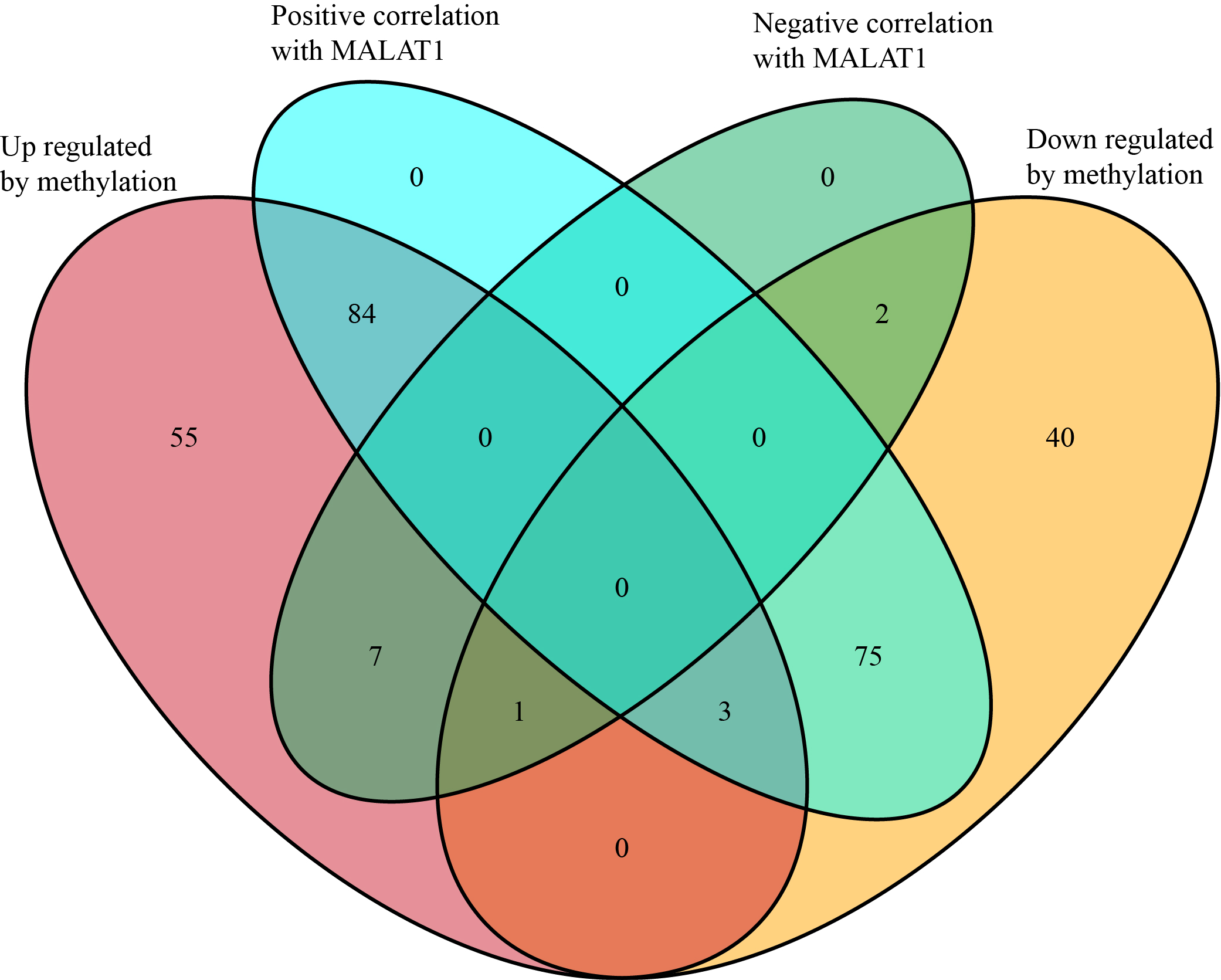

Supplement: Supplementary file 5 [file Image5.JPEG]
